# Ubiquitination Regulates Reorganization of the Membrane System During Cytomegalovirus Infection

**DOI:** 10.3390/life15081212

**Published:** 2025-07-31

**Authors:** Barbara Radić, Igor Štimac, Alen Omerović, Ivona Viduka, Marina Marcelić, Gordana Blagojević Zagorac, Pero Lučin, Hana Mahmutefendić Lučin

**Affiliations:** 1 Department of Physiology, Immunology and Pathophysiology, Faculty of Medicine, University of Rijeka, Braće Branchetta 20, 51000 Rijeka, Croatia; barbara.radic@uniri.hr (B.R.); igor.stimac@uniri.hr (I.Š.); alen.omerovic@uniri.hr (A.O.); ivona.viduka@uniri.hr (I.V.); mmarcelic@uniri.hr (M.M.); gordana.blagojevic@uniri.hr (G.B.Z.); 2 Campus University Center Varaždin, University North, Jurja Križanića 31b, 42000 Varaždin, Croatia

**Keywords:** cytomegalovirus, assembly compartment, secondary envelopment, ubiquitination, PYR-41, WASH, Rab10, tubular recycling endosomes

## Abstract

Background: During infection with the cytomegalovirus (CMV), the membrane system of the infected cell is remodelled into a megastructure called the assembly compartment (AC). These extensive changes may involve the manipulation of the host cell proteome by targeting a pleiotropic function of the cell such as ubiquitination (Ub). In this study, we investigate whether the Ub system is required for the establishment and maintenance of the AC in murine CMV (MCMV)-infected cells Methods: NIH3T3 cells were infected with wild-type and recombinant MCMVs and the Ub system was inhibited with PYR-41. The expression of viral and host cell proteins was analyzed by Western blot. AC formation was monitored by immunofluorescence with confocal imaging and long-term live imaging as the dislocation of the Golgi and expansion of Rab10-positive tubular membranes (Rab10 TMs). A cell line with inducible expression of hemagglutinin (HA)-Ub was constructed to monitor ubiquitination. siRNA was used to deplete host cell factors. Infectious virion production was monitored using the plaque assay. Results: The Ub system is required for the establishment of the infection, progression of the replication cycle, viral gene expression and production of infectious virions. The Ub system also regulates the establishment and maintenance of the AC, including the expansion of Rab10 TMs. Increased ubiquitination of WASHC1, which is recruited to the machinery that drives the growth of Rab10 TMs, is consistent with Ub-dependent rheostatic control of membrane tubulation and the continued expansion of Rab10 TMs. Conclusions: The Ub system is intensively utilized at all stages of the MCMV replication cycle, including the reorganization of the membrane system into the AC. Disruption of rheostatic control of the membrane tubulation by ubiquitination and expansion of Rab10 TREs within the AC may contribute to the development of a sufficient amount of tubular membranes for virion envelopment.

## 1. Introduction

Human cytomegalovirus (HCMV) is an important pathogen that infects a large proportion of the human population [1] and is associated with many pathophysiological conditions [2], severe infections in immunocompromised individuals [3] and birth defects in infants [4]. It enters into a complex interaction with the infected host and leads to different outcomes in infected cells [5]. Lytic infection is accompanied by a complete reorganization of the infected cell, including the establishment of a new organelle structure of the cell’s membrane system [6]. This complex interaction is favoured by the relatively high coding potential of the large cytomegalovirus (CMV) genome [7,8,9]. The obstacle in studying the interaction of the HCMV with infected humans is bridged by using murine CMV (MCMV) as a model for many aspects of CMV pathogenesis, including interaction with cellular processes and the reorganization of infected cells [10].

CMV infection involves the interaction with host cell systems, beginning with virion entry, establishment of infection, integration of viral DNA, expression of CMV genes, interference with host cell signalling processes that promote viral gene expression, reorganization of the nucleus and membrane system to establish the virus assembly line and reconstruction of membrane organelles to establish the egress pathway [5,6,11,12,13]. Infection is accompanied by a massive reorganization of the nucleus and cytoplasm of the infected cell [14] by the formation of two megastructures, the nuclear replication centres (NRCs) and the assembly compartments (ACs) [6].

In MCMV-infected fibroblasts and fibroblast-like cells, reorganization is initiated very early during infection [15,16,17,18,19]. The MCMV coding programme is activated immediately after viral DNA integration, leading to the rapid expression of α genes, termed immediate-early (IE) genes, within the first hour [7]. In the second hour post-infection, IE proteins initiate the expression of the first set of early (E) genes known as β1 genes, the products of which regulate and tune the expression of two waves of the second set of E genes, the “maintained early (β2)” genes at 3–4 h post-infection (hpi) and the “delayed early (β3)” genes at 6–7 hpi [7]. The β1 and β2 gene products drive the formation of NRCs [14,20] and pre-ACs [15,17] in the E phase of infection and form the machinery for viral DNA replication [7], which occurs at 15–16 hpi [21]. The replicated viral DNA is required for the expression of 228 late phase (L) transcripts, which can be divided into “canonical late (γ1)” genes, initiated at 16–18 hpi, and “delayed late (γ2)” genes, initiated gradually after 24 hpi [7]. The products of the γ1 and γ2 genes load the pre-AC, which mature during the E phase of infection through a reorganization of the membrane system into the cytoplasmic virion factory known as the AC [15].

The basic configuration of the AC is identical in HCMV- and MCMV-infected cells [15,22,23]. It involves the uncoupling and relocation of the Golgi into a large ring-like configuration that encloses a large collection of membranous organelles that include early endosomes (EEs), recycling endosomes (REs), the endosomal recycling compartment (ERC), the trans-Golgi network (TGN) and expanded intermediates between these compartments [15,22,24]. In MCMV-infected cells, the reorganization of the membrane system occurs rapidly in the E phase of infection, and the basic configuration of the AC is established after 6–8 hpi [15,17,18], suggesting that these processes are initiated by β2 or β3 genes. The structure that emerges in the E phase of infection prior to viral DNA replication and the expression of L genes is termed pre-AC. The predominant feature of pre-AC is an extensive tubulation of EEs and ERC, as evidenced by the accumulation of Rab10-positive tubular membranes (Rab10 TMs) or Evectin2-positive membranes of the ERC [15,17,18]. The accumulation of several membrane organelle markers associated with tubulation, such as Arf1, Arf6, Rab35 and Rab15 [15,25], suggests that multiple tubular membrane intermediates are expanded within the pre-AC. As shown by live imaging of Rab10 TMs, these structures grow and shrink very dynamically [18]. These properties can be utilized in the final stages of CMV assembly, the so-called secondary envelopment, which comprises the membrane-enveloping of biomolecular condensates of tegument proteins loaded with nucleocapsids to generate infectious virions [13].

The establishment of the basic configuration of pre-AC in MCMV-infected cells is a function of one or more E genes belonging to either the β2 or β3 temporal subset. These subgroups comprise 89 transcripts whose products build the viral DNA replication machinery, modulate host immune functions and reorganize cell structure, including membranous organelles [7]. Nevertheless, the fraction of the coding potential that could be involved in the reorganization of the infected cell is not very large, as high-throughput studies in HCMV-infected cells [26,27] have shown that more than a thousand host cell proteins are affected by the infection. Therefore, it can be assumed that this limited coding potential targets some general cellular processes that maintain the intracellular homeostasis of the membrane system. The proteome of the infected cell can be reorganized by influencing the transcriptional activity of host cell genes, as has been shown in several studies in HCMV [28,29] and MCMV [15,21,30] infections. In addition, the proteome of the infected cell can be remodelled by the ubiquitination (Ub) of host cell proteins [31,32,33], either to regulate protein levels by diverting them into the degradation pathway or by modulating protein activity within the cellular rheostat mechanism. The Ub mechanism can also modulate the expression of viral proteins, as shown in a study on HCMV-infected cells [31,34,35,36].

The aim of this study is to investigate whether the Ub system is required for the reorganization of the membrane system and the establishment and maintenance of the AC in MCMV-infected cells. The answer to this question is necessary for the further development of approaches to identify the mechanistic basis of the AC establishment and maintenance and the regulation of the secondary envelopment process. We have taken the approach of inhibiting the Ub system with PYR-41 (4[4-(5-nitro-furan-2-ylmethylene)-3,5-dioxo-pyrazolidin-1-yl]-benzoic acid ethyl ester), the irreversible and cell-permeable inhibitor of the ubiquitin-activating enzyme (E1) [37]. Since E1 activation is the first step of ubiquitination and only a single essential E1 protein has been detected in humans [38,39], PYR-41 is expected to strongly affect the ubiquitination of the proteome in treated cells. We show that the Ub system is intensively utilized at all stages of the MCMV replication cycle, including the reorganization of the membrane system into AC. Disruption of rheostatic control of membrane tubulation by the ubiquitination and expansion of Rab10 TMs within the AC may contribute to the development of a sufficient amount of tubular membranes for virion envelopment.

## 2. Materials and Methods

### 2.1. Cell Lines and Cell Culture

Murine fibroblast-like cell line NIH3T3 (ATCC CRL-1658) was used for most experiments, and murine embryonic fibroblasts (MEFs), derived from 17-day-old BALB/c mouse embryos, were used for virus production and plaque assays. The NIH3T3 EGFP-Rab10wt cell line [17] was used for digital holotomographic microscopy (DHTM) experiments. Cells were propagated in 10 cm Petri dishes with Dulbecco’s Modified Eagle’s Medium (DMEM) supplemented with fetal bovine serum (FBS) (10% for NIH3T3 cells, 5% for MEFs), 2 mM L-glutamine, 100 mg/mL streptomycin and 100 U/mL penicillin (all reagents from Gibco/Invitrogen, Grand Island, NY, USA). When the cells were 80–85% confluent, they were divided and used for experiments.

### 2.2. Viruses and Infection Conditions

Unless otherwise stated, wild-type (wt) MCMV (Smith strain, ATCC VR-194) was used. The recombinant virus Δm138-MCMV (ΔMC95.15), with the deleted m138 (fcr1) gene [40], was used in the immunofluorescence microscopy experiments when mouse IgG_2a_ antibodies were used to avoid FcR-mediated non-specific binding, as previously described [18]. C3X-GFP-MCMV (MCMV-GFP) was used for flow cytometric detection of the establishment of the MCMV infection, where the GFP gene was inserted upstream of the ie2 gene of the wt MCMV background under the control of the HCMV major IE enhanced promoter (MIEP) [41].

The production of MCMV stocks and the infection of mouse fibroblasts was carried out according to the standard protocol [42]. In all experiments, the cells were infected with an MOI (Multiplicity of Infection) of 10 (1 PFU/cell). The expression of the immediate-early 1 protein (pIE1) was used to verify infection in the tested cells or cell extracts as previously described [15].

### 2.3. Antibodies and Reagents

The following monoclonal (mAb) or polyclonal (pAb) antibodies against host cell proteins were used: Rabbit mAb (IgG) against Rab10 (Cell Signaling Inc., Danvers, MA, USA; Cat. No. 8127), mouse mAb (IgG_1_) against GM130 (Cat.No. 610823, BD Biosciences, Franklin Lakes, NJ, USA), mouse mAb (IgG) against WASHC1 (Sigma-Aldrich Chemie GmbH (Schnelldorf, Germany) Cat. No. SAB4200552), mouse mAb against β-actin (IgG_1_, Millipore, Burlington, MA, USA; Cat. No. MAB150) and mouse mAb (IgG_2a_) against β-tubulin (Proteintech Group, Inc., Rosemond, IL, USA, Cat. No. 66240-1-Ig). Rabbit pAb (IgG) against hemagglutinin (HA) (Proteintech Group, Inc., Rosemond, IL, USA, Cat. No. 51064-2-AP) was used to detect HA-ubiquitin fusion protein.

The expression of MCMV proteins was monitored using mAbs produced, purified and verified by the University of Rijeka Center for Proteomics, (https://products.capri.com.hr/shop/?swoof=1&pa_reactivity=murine-cytomegalovirus; accessed on 28 June 2025). The following antibodies were used: mouse mAb (IgG_1_, clone CROMA101) and (IgG_2a_, clone IE1.01) against pm123/pIE1, mouse mAb (IgG_1_, clone CROMA103) against pM112-113 (E1), mouse mAb against pM57 (clone M57.02), mouse mAb (IgG_1_, clone 74.01) against pM74.

Polyclonal secondary antibodies for immunofluorescence and confocal microscopy were as follows: Alexa Fluor (AF)^488^- and AF^555^-conjugated (Molecular Probes; Leiden, The Netherlands) and AF^680^-conjugated (Jackson ImmunoResearch, West Grove, PA, USA) goat antibodies against mouse IgG_1_, mouse IgG_2a_ and rabbit IgG primary antibodies. Goat anti-mouse and goat anti-rabbit antibodies conjugated to HRP were used as polyclonal secondary antibodies for Western blotting (Jackson ImmunoResearch, West Grove, PA, USA).

DAPI (4,6-diamidino-2-phenylindole dihydrochloride) was purchased from Thermo Fisher Scientific (Waltham, MA, USA; Cat. No. D1306). Puromycin was obtained from Santa Cruz Biotechology Inc., Dallas, TX, USA. Propidium iodide was from Sigma-Aldrich Chemie GmbH (Schnelldorf, Germany).

### 2.4. Inhibition of Ubiquitination

PYR-41 (4-[4-[(5-nitro-2-furanyl)methylene]-3,5-dioxo-1-pyrazolidinyl]benzoic acid ethyl ester), obtained from Sigma-Aldrich Chemie GmbH (Schnelldorf, Germany; Cat. No. 000135538), was dissolved in DMSO (Sigma-Aldrich Chemie GmbH (Schnelldorf, Germany). When the PYR-41 effect was tested, the control (mock treated) cells were incubated with the corresponding DMSO concentration (0.04% DMSO for 10 μM, 0.06% DMSO for 15 μM and 0.08% DMSO for 20 μM PYR-41).

### 2.5. Flow Cytometry for Detection of Establishment of Infection with C3X-GFP-MCMV

NIH3T3 cells were infected with C3X-GFP-MCMV (MOI of 10) and treated with PYR-41 (10 μM, 15 μM, or 20 μM) at the indicated time points. Samples were taken at 0 hpi and 6 hpi and the intensity of the GFP signal and the percentage of GFP-positive cells were quantified by flow cytometry (FACSCalibur flow cytometer; Becton Dickinson & Co, San Jose, CA, USA), as previously published [17]. Only viable (propidium iodide-negative) cells were analyzed (5000 cells in total).

### 2.6. Immunofluorescence, Confocal Microscopy and Image Analysis

The cells were cultured in 12- or 24-well plates until they were 60–70% confluent. After treatment according to the experimental protocols, the cells were fixed (4% paraformaldehyde for 20 min at r.t.), permeabilized (0.5% Tween 20 for 20 min at 37 °C), incubated with the appropriate primary and secondary antibodies (60 min at 37 °C) and embedded in Mowiol (Fluka Chemicals, Selzee, Germany)-DABCO (Sigma Chemical Co., Steinheim, Germany) in PBS containing 50% glycerol. The samples were analyzed by immunofluorescence and confocal microscopy.

The inverted confocal microscope Leica DMI8 (Leica Microsystems GmbH, Wetzlar, Germany) was equipped with a confocal section: TCS SP8, 4 lasers (UV (Diode 405) for DAPI, Ar 488 for AF488, DPSS 561 for AF555 and He/Ne 633 for AF680) and 4 detectors (2 PMT and 2 HyD). The HC PLAPO CS2 objective (63 × 1.40 oil) was used for all experiments. The images were acquired with the software LAS (Leica Application Suite) X version 3.5.6.21594 (Leica Microsystems GmbH, Wetzlar, Germany). The sequential mode with a z-series of 0.5 μm was used. The parameters were not changed in the experiment for the samples compared.

The percentage of pIE1-positive cells and the perinuclear accumulation of host cell proteins in pre-AC or AC were quantified directly using an Olympus BX51 epifluorescence microscope with a DP71 CCD camera (Olympus, Tokyo, Japan). The UPlanFL N 40×/0.75 objective was used as previously described [19]. In brief, the concentrated perinuclear signal within an angle of α ≤ 90° was considered as pre-AC or AC. At least 10 image fields were analyzed for each microscopic sample (approximately 200–400 cells per sample).

### 2.7. Time-Lapse Acquisitions Using Holotomographic Microscopy

DHTM in combination with epifluorescence was performed using the 3D Cell-Explorer Fluo (Nanolive, Ecublens, Switzerland) with a ×60 air objective at a wavelength of λ = 520 nm. Fluorescence excitation is provided by a CoolLED pE-300 Ultra with standard DAPI/FITC/TRITC filters. The irradiance of the laser was 0.2 nW/μm^2^, and the acquisition time per image was 45 ms. An incubator (Okolab, Pozzuoli, Italy) with a heated glass lid to avoid condensation was used to maintain physiological conditions during live cell imaging, including a constant temperature of 37 °C, 90% relative humidity and 5% CO_2_ concentration. For imaging, 100,000 cells infected with MCMV (MOI of 10) were plated in µ-Slide I Luer (Ibidi GmbH, Gräfelfing, Germany) with surface modification ibiTreat and channel height 0.8 mm. The channel slide was placed in the incubation chamber for acclimatization prior to imaging. Imaging started 16 h after infection and the acquisition time per image was every ten seconds for one hour. PYR-41 was added in the channel one minute after starting the acquisition. Time-lapse images were saved and exported as 2D TIFF format using STEVE software, version 1.6.3496 (Nanolive SA, Ecublens, Switzerland), which controls the 3D Cell Explorer microscope. Exported images were merged and converted in avi. format using FIJI [43].

### 2.8. Western Blot Analysis

Whole cell lysates (WCLs) were prepared after lysing the cells with the RIPA buffer (Thermo Fisher Scientific (Waltham, MA, USA; Cat. No. D1306) supplemented with protease and phosphatase inhibitors (Roche Diagnostics GmbH, Unterhaching, Germany; Cat. No. 11697498001) and mixed with a sample buffer (50% glycerol, 10% SDS, 0.05% bromophenol blue, 0.3 M Tris, pH 6.8). Samples were incubated at 96 °C (5 min), separated by SDS-PAGE and blotted onto a polyvinylidene difluoride membrane (PVDF-P WB membrane; Millipore, Burlington, MA, USA). After 2 h of blotting at 80 V (Bio-Rad Trans-Blot Turbo Transfer System; Hercules, CA, USA), the membranes were blocked for 2 h in 1% blocking reagent (Roche Diagnostics GmbH, Mannheim, Germany) and incubated with the corresponding primary antibody (overnight at 4 °C). After washing three times in T-TBS (TBS with 0.05% Tween 20; pH = 7.5) and incubation with peroxidase-conjugated secondary antibodies (dissolved in 0.5% blocking reagent with T-TBS for 1 h), the membranes were finally washed in T-TBS. The intensity of the chemiluminescent signal was measured with chemiluminescence (SignalFire [TM] Plus ECL reagent or SignalFire [TM] Elite ECL reagent; Cell Signaling, Cat. No. 12630S or 12757P) with ImageQuant LAS 500 (GE Healthcare Bio-Sciences AB, Upsala, Sweden). Expression of the protein of interest was visualized together with the loading control (β-actin or β-tubulin) on the same membrane for each experiment. Quantification of the detected signals was performed using ImageJ 1.53 software and ImageQuantTL, version 10.2, Cytiva, with all values normalized to the loading controls. The following formulas were used. (1) Normalization of loading control signal (β-actin/β-tubulin): lane normalization factor = observed actin signal for each lane/highest observed β-actin/β-tubulin signal for the blot, and (2) normalization of experimental signal: raw signal value/normalized index of β-actin or β-tubulin. The effect of PYR-41 on the expression of the MCMV protein of interest was represented as a fold change in signal expression compared to the band with the highest intensity during MCMV kinetics in mock-treated samples.

### 2.9. siRNA Silencing of WASHC1 and Rab11

Small interfering (si)RNA for WASHC1 (Cat. No. L-054931-01-0005) was from Dharmacon Inc. (Laffayete, CO, USA), Mm_Rab11_1 sequence (s100211729) was from Qiagen (Hilden, Germany), and non-targeting negative siRNA (Cat. No. 1022076) was from Qiagen (Hilden, Germany). The RNAiMAX-Lipofectamine reagent (Invitrogen, Carlsbad, CA, USA) was mixed with the siRNA WASHC1 (80 nM), siRNA Rab11 (20 nM) or the non-targeted negative siRNA according to the manufacturer’s guidelines and added dropwise to the cells. After 6 h, the cell culture medium was replaced with DMEM containing 10% FCS, incubated for a further 40 h and then the experiment was performed.

### 2.10. Subcloning of HA-Ubiquitin Sequence into the Lentiviral Vector pLIX-Kan_PstI and Generation of the NIH3T3-HA-Ub Cell Line

HA-ubiquitin (HA-Ub) from the pRK5-HA-ubiquitin-WT plasmid, a gift from Ted Dawson (Addgene plasmid #17608; http://n2t.net/addgene:17608, accessed on 27 July 2025; RRID:Addgene_17608) [44], was subcloned into the lentiviral vector pLIX402_Kan, which enables the doxycycline-inducible expression of the transgene. The lentiviral vector pLIX402_Kan_PstI (a gift from dr. Martin Messerle (Hannover, Germany) [45] was derived from pLIX402 (Addgene plasmid # 41394) by insertion of a kanamycin cassette. The primers for PCR amplification of the HA-Ub ORF (F-HA-Ub: 5′-CGCCTGGAGAATTGGCTGCAGCCCGAACCGACAGTCGGT-3′ and R-HA-Ub: 5′-AAGGCGCAACCCCAACCCCGTCAACCACCTCTGAGACGG-3′) were designed according to the original plasmids. After pLIX402_Kan_PstI was cleaved with the restriction endonucleases PstI (Cat. No. R3140S, New England Biolabs Inc., Ipswich, MA, USA) and BamHI (Cat. No. R3136S, New England Biolabs Inc., Ipswich, MA, USA), the kanamycin cassette was removed and replaced with PCR-amplified Ha-Ub ORF. The NEBuilder HiFi DNA Assembly Cloning Kit (New England Biolabs (NEB) Mass, Ipswich, MA, USA) was used according to the manufacturer’s instructions.

After verification, the pLIX HA-Ub plasmid was used to produce lentiviruses. Briefly, 5 μg of the pLIX HA-Ub lentiviral plasmid was mixed with 10 μg of p8.91 (a gift from Simon Davis; Addgene plasmid # 187441; http://n2t.net/addgene:187441, accessed on 27 July 2025; RRID:Addgene_187441) [46] and 0.5 μg of plasmid p-CMV-VSV-G (a gift from Bob Weinberg; Addgene plasmid #8454; http://n2t.net/addgene:8454, accessed on 27 July 2025; RRID:Addgene_8454) [47], in 1.5 mL Optimem (Thermo Fisher Scientific, Waltham, MA, USA; (Cat. No. 31-985-070). In parallel, 41 μL Lipofectamine 3000 was dissolved in 1.5 mL Optimem; both solutions were mixed and added dropwise to HEK 293T cells (ATCC CRL-3216, Manassas, VA, USA) cultured in DMEM with 10% FCS but without antibiotics. Lentiviruses were recovered from the supernatants (24, 30 and 48 h after transfection) after centrifugation (5 min at 2000 rpm) and filtration (0.45 μM filter) and used to transduce NIH3T3 cells to produce the NIH3T3 HA-Ub cell line. The HA-Ub-expressing cells were selected with puromycin (2.5 μg/mL).

### 2.11. Immunoprecipitation of Ubiqutinated Proteins in the NIH3T3 HA-Ub Cell Line

MCMV-infected (16 hpi) and non-infected cells were lysed in 200 µL of 1% NP40 lysis buffer containing 20 mM Tris–Cl, (pH 7.6), 140 mM NaCl, 5 mM MgCl2, 25x complete protease inhibitor cocktail (Roche Diagnostics GmbH, Unterhaching, Germany; Cat. No. 11697498001) and 1 mM PMSF (Sigma, Cat. No. P7626-5G). Quantitative immunoprecipitation of proteins with conjugated HA-Ub was performed with rabbit pAb against hemagglutinin (HA) (Proteintech Group, Inc., Rosemond, IL, USA, Cat. No. 51064-2-AP) (2 µg/mL) (1 h, 4 °C). Immunocomplexes of HA-Ub-conjugated proteins were retrieved with Protein A-Sepharose (PAS) (Cytiva Sweden AB, Uppsala, Sweden, Cat. No. 17078001). After the elution of the immune complexes at 96 °C in the sample buffer, the proteins were analyzed by Western blot.

### 2.12. Viral Growth and Plaque Assay

After infection with MCMV at an MOI of 10, cell culture supernatants and cell lysates were collected through two freeze/thaw cycles at 0, 24, 48, 72 and 96 hpi to establish a one-step growth curve of MCMV. The released (supernatant) and cell-associated infectious virions were determined using the standard plaque assay as previously described [19].

### 2.13. Statistical Analysis and Data Presentation

Data are presented as individual values from separate experiments and as mean ± standard deviation (SD). Statistical significance was determined for all experiments using a two-tailed Student’s *t*-test (Excel software, Microsoft, Redmond, WA, USA), with the exception of the plaque assays, for which the Mann–Whitney (U) test (MedCalc, version 19.7.2) was used. The statistical difference was indicated by the *p*-value (* *p* < 0.05; ** *p* < 0.01, *** *p* < 0.001).

## 3. Results

### 3.1. Ubiquitination in the Establishment of MCMV Infection and Progression of the Replication Cycle

To examine the effect of PYR-41 on the establishment and maintenance of the AC, it was first essential to investigate the effect of PYR-41 on the establishment of MCMV infection, the progression of the MCMV replication cycle and the overall effect on virion production.

#### 3.1.1. Inhibition of Ubiquitination Prevents Establishment of MCMV Infection

We treated NIH3T3 cells with different concentrations of PYR-41 (10, 15 and 20 μM) and tested their effect on the establishment of infection using two approaches: infection with the recombinant MCMV C3X-GFP-MCMV (MCMV-GFP), which expresses GFP, and expression of pIE1, a product of the α-gene m123, as an indication of successful integration of the MCMV genome into the infected cell.

**Figure 1:**
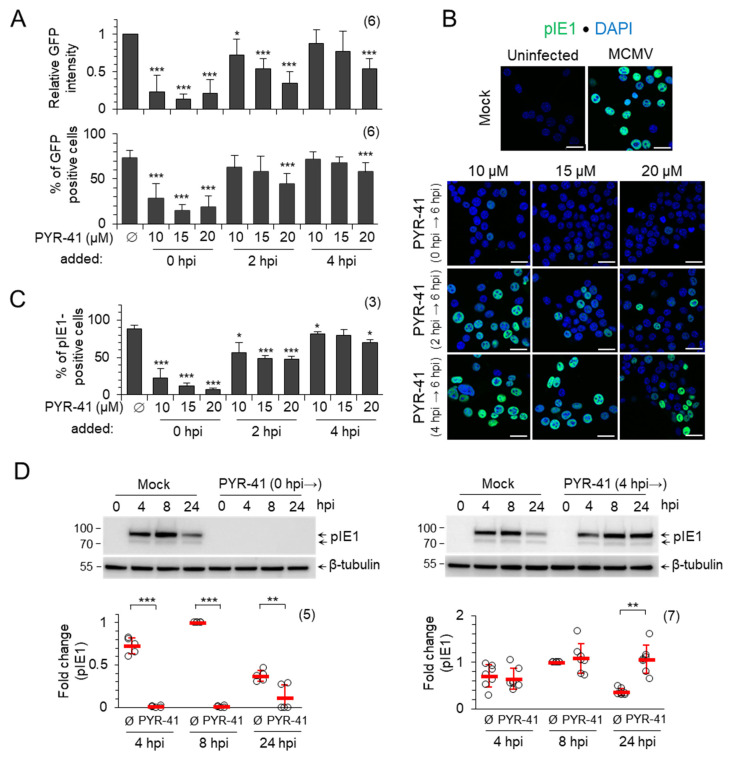
PYR-41-inhibited ubiquitination prevents the establishment of the MCMV infection. (**A**) NIH3T3 fibroblasts were infected with C3X GFP MCMV and treated with DMSO (Ø) or indicated concentrations of PYR-41 at 0, 2 and 4 hpi. GFP expression was determined by flow cytometry after 6 hpi (Figure S1). The upper panel shows GFP intensity normalized to mock-treated cells, and the lower panel shows the percentage of GFP-positive cells. (**B**) pIE1 expression after 6 hpi and (**C**) percentage of pIE1-positive cells (mean ± SD) after infection with MCMV and treatment with DMSO (mock) or different concentrations of PYR-41 at 0, 2 and 4 hpi. Bars—25 μm. (**D**) MCMV-infected cells were treated with 15 μM PYR-41 before (0 hpi →) or 4 h after infection (4 hpi →) and the expression of pIE1 was determined by Western blot analysis. Shown are representative Western blots (complete blots are shown in Figure S4) and a quantitative analysis of the signals normalized to β-tubulin. The fold changes represent the signal expression relative to the band with the highest intensity in the mock-treated (Ø) samples, the read bars represent the mean ± SD, and the empty circles are data from an independent experiment. Statistical significance was determined using a two-tailed paired Student *t*-test (*** *p* < 0.001; ** *p* < 0.01; * *p* < 0.05). The numbers of independent experiments are indicated in parenthesis.

Infection of cells with C3X-GFP-MCMV (MCMV-GFP), in which the GFP gene was inserted under the control of the HCMV-MIEP (major IE enhanced promoter) and upstream of the ie2 gene [41], resulted in the expression of GFP in the infected cell, which can be monitored and quantified at the single cell level by flow cytometry (Figure S1). The treatment of the cells with the three concentrations of PYR-41 simultaneously with infection (0 hpi) resulted in the almost complete abrogation of GFP expression (Figure 1A, upper panel), as determined by the flow cytometric quantification of the profiles after 6 hpi (Figure S1).

A weak fluorescent signal can be detected in a small fraction of cells (between 15 and 28% of cells; lower panel in Figure 1A and Figure S1). These data indicate that the functional ubiquitination system of the host cell is required for the establishment of the infection and the initiation of MCMV genome transcription. The addition of PYR-41 at 2 hpi, prior to the expression of the β2 genes, still resulted in the concentration-dependent decrease in GFP expression (Figure 1A and Figure S1), suggesting that the inhibition of the Ub system at this stage still blocks the expression of IE phase genes and progression into the E phase of infection. The addition of PYR-41 at 4 hpi, after the expression of β2 genes, had very little effect on GFP expression at 10 and 15 μM concentration (Figure 1A and Figure S1). The 20 μM concentration had an effect on GFP expression in viable cells (Figure 1A and Figure S1); however, almost half of the cells incorporated propidium iodide, suggesting borderline concentration in short-term experiments.

Considering the known heterogeneity of NIH3T3 cells [48] and the non-synchronicity in the progression through the E phase of infection, we also analyzed pIE1 expression in PYR-41-treated cells by immunofluorescence (Figure S2). In mock-treated cells, pIE1 was detected as a nuclear signal in 90% of the cells, which is consistent with the infection level observed in previous studies at an MOI of 10 [15,20]. Treatment of cells with PYR-41 at 2 hpi, but not at 4 hpi, decreased the proportion of pIE1-positive cells (Figure 1B,C), consistent with the observed effect of PYR-41 on IE gene expression (Figure 1A). Overall, these data suggest that the disruption of ubiquitination after 4 hpi has little effect on MCMV IE gene expression.

Based on these data, we used a PYR-41 concentration of 15 μM, which preserved cell viability up to 48 hpi (Figure S3), to test the expression of pIE1 by Western blot and immunofluorescence. In mock-treated cells, the expression of pIE1 reached a plateau at 8 hpi and decreased at 24 hpi (Figure 1D, upper panel), which is consistent with the known kinetics of pIE1 expression in NIH3T3 cells [7,21]. The treatment of cells with 15 μM PYR-41 simultaneously with infection (0 hpi) resulted in an almost complete loss of pIE1 expression, as shown by the representative Western blot (Figure 1D, left) and quantitative analysis of five independent experiments (Figure 1D, left). However, when the cells were treated with 15 μM PYR-41 at 4 hpi, the expression of pIE1 at 8 hpi was similar to that of mock-treated cells, but increased sharply at 24 hpi (Figure 1D, right), suggesting that ubiquitination is a mechanism controlling pIE1 levels in infected cells.

#### 3.1.2. Ubiquitination Regulates pIE1 Function

**Figure 2:**
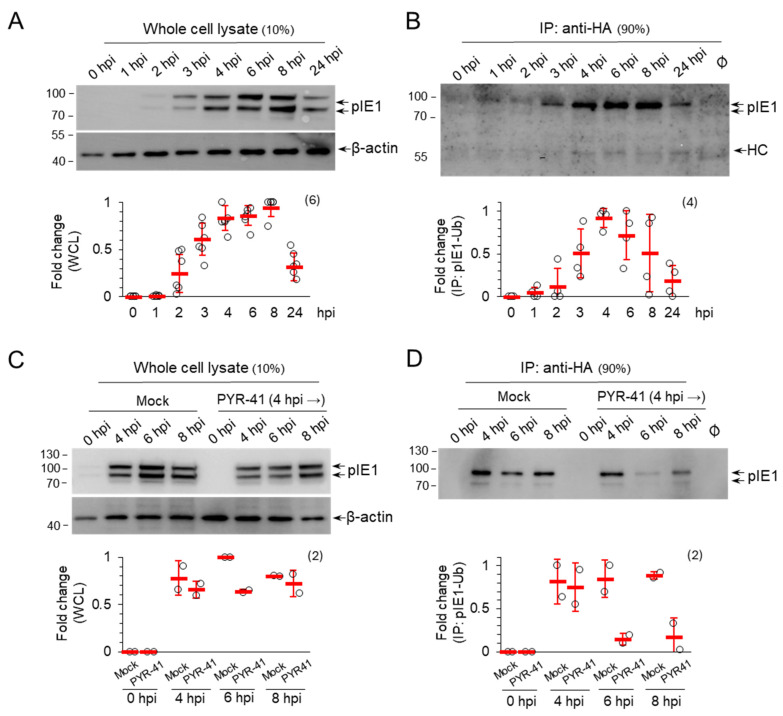
pIE1 is ubiquitinated in MCMV-infected cells. Doxycycline-treated (2 μg/mL for 48 h) NIH3T3 HA-Ub cells were infected with MCMV and (**A**,**B**) were left untreated or (**C**,**D**) treated with either DMSO (mock) or 15 μM PYR-41 at 4 hpi. At indicated times after infection, the whole cell lysates (**A**,**C**) and anti-HA immunoprecipitate (**B**,**D**) were analyzed for pIE1 and β-actin expression by Western blot. Signals were quantified by ImageJ and normalized to β-actin in WCLs. Fold changes represent the signal expression relative to the band with the highest intensity in mock-treated cells. The mean ± SD (red bars) and individual data (empty circles) are shown in the graphs. The number of independent experiments is indicated in parenthesis. The original blots are shown in Figure S6.

Because the Western blot analysis of pIE1 expression suggests that ubiquitination may be a mechanism controlling the amount of pIE1 in infected cells, we next investigated whether pIE1 is ubiquitinated. We generated the NIH3T3 cell line with the inducible expression of HA-Ub (NIH3T3 HA-Ub) (Figure S5), infected these cells with MCMV after 48 h induction with doxycycline, and performed temporal analysis of pIE1 expression by Western blot either directly from cell lysates (Figure 2A) or after the immunoprecipitation of lysates by rabbit anti-HA antibody (Figure 2B). pIE1 was already detected at 2 hpi, its level gradually increased as the E phase progressed and decreased to 10–20% of the maximal signal at later stages of infection (Figure 2A). After immunoprecipitation of HA-Ub tagged proteins, ubiquitinated pIE1 was detected as early as 3 hpi and remained continuously ubiquitinated throughout the E phase (Figure 2B), indicating that the infected cell regulates either the amount or the function of pIE1 by ubiquitination. Treatment of the cells with PYR-41 at 4 hpi did not increase the amount of pIE1 (Figure 2C) and inhibited its ubiquitination (Figure 2D), suggesting that ubiquitination may be used to regulate the function of pIE1 or its localization, which may be an important mechanism required for the normal progression of the MCMV replication cycle. Unfortunately, due to the lack of specific antibodies, we were unable to test the pIE3 protein, which is known to control the onset of MCMV gene activation [7,41].

#### 3.1.3. Ubiquitination Is Required for Progression of MCMV Replication Cycle

**Figure 3:**
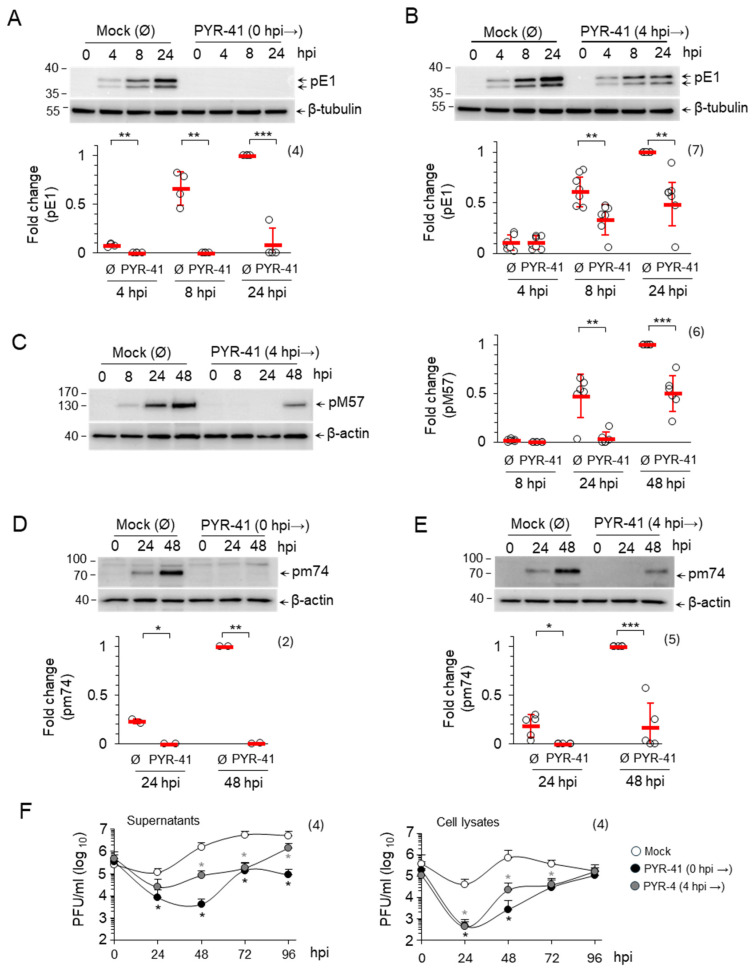
The effect of PYR-41 on the progression of the MCMV replication cycle. (**A**–**E**) MCMV-infected NIH3T3 cells were treated with either DMSO (mock) or with 15 μM PYR-41 prior to infection (0 hpi) or 4 hpi. The expression of pE1 (**A**,**B**), pM57 (**C**) and pM74 (**D**,**E**) was analyzed by Western blot at the indicated time points after infection. β-tubulin or β-actin were used as loading controls. The original blots are shown in Figures S4 and S7. Signals were normalized to the loading control, and fold changes represent signal expression relative to the band with the highest intensity in the mock-treated samples. The empty circles show the data from independent experiments and the red bars show mean values ± SD. Statistical significance was determined using a two-tailed paired Student *t*-test (*** *p* < 0.001; ** *p* < 0.01; * *p* < 0.05). The number of independent experiments is indicated in parenthesis. (**F**) Supernatants or cell lysates of PYR-41-treated cells (0 hpi → or 4 hpi →) were harvested at 0, 24, 48, 72 and 96 hpi and the number of infectious units was determined by plaque assay. The mean values ± SD of four independent experiments are shown. Statistical significance was determined using the Mann–Whitney test (* *p* < 0.05).

The effect of 15 μM PYR-41 treatment at 0 and 4 hpi on the progression of the MCMV replication cycle was analyzed by Western blot quantification of representative MCMV proteins: pE1, an organizer of NRCs which is a product of the m112 gene belonging to the β1-temporal subgroup of E genes, pM57, a major DNA-binding protein encoded by the M57 gene belonging to the β3-temporal subgroup of E genes and pm74, a viral glycoprotein which is a product of the m74 gene belonging to the γ1-temporal subgroup of L genes [7,49]. In the mock-treated cells, pE1 was detected at 4 hpi and accumulated as the E phase progressed (8 hpi) towards the onset of the L phase (24 hpi), pM57 was detected at 8 hpi and accumulated at the onset of the L phase (24 hpi) and in the advanced L phase (48 hpi), while pm74 was not detected in the E phase but accumulated in the L phase (Figure 3A–E, mock treated). In agreement with our observation above (Figure 1), treatment of the cells with PYR-41 at 0 hpi prevented the expression of the viral proteins, as shown by the analysis of pE1 (Figure 3A) and pm74 (Figure 3D). However, PYR-41 treatment at 4 hpi did not prevent the expression of pE1 (Figure 3B), pM57 (Figure 3C) and pm74 (Figure 3E), but strongly decreased their levels in infected cells compared to the mock-treated controls (Figure 3B,C,E). These data suggest that ubiquitination is essential for regulating MCMV gene expression and progression through the replication cycle and are consistent with previous conclusions on its role in regulating IE proteins.

Reduced viral gene expression should lead to the lower production of virions. Therefore, we performed a standard one-step growth analysis of virion production using the plaque assay. Inhibition of ubiquitination before the infection resulted in a drastic reduction in both cell-associated and released infectious virions. After 24 hpi, almost no infectious units could be detected in the cell lysate and only very few in the supernatant. After 48 hpi, the amount of assembled infectious units in the cells and of released units was about 300 times lower than in mock-treated cells (Figure 3F). At later time points, the amount of assembled infectious units in the infected cells gradually increased and reached the level of mock-treated cells after 96 hpi (Figure 3F, cell lysates). A similar effect was observed when the cells were treated with PYR-41 at 4 hpi, although resulting in a 12-fold reduction in infectious virions. These data suggest that ubiquitination is required for the assembly and release of infectious virions (Figure 3F). Although the amount of available viral proteins in the infected cells is reduced, it is sufficient to form new virions as an excess of viral compounds. These quantitative discrepancies, particularly the difference in one logarithm of infectious units in the cells and in the supernatant, therefore, suggest that ubiquitination also contributes to the cellular processes involved in the formation and release of virions.

### 3.2. Ubiquitination in the Reorganization of Membranous System into AC

#### 3.2.1. Ubiquitination Is Required for Establishment of the Pre-AC

**Figure 4:**
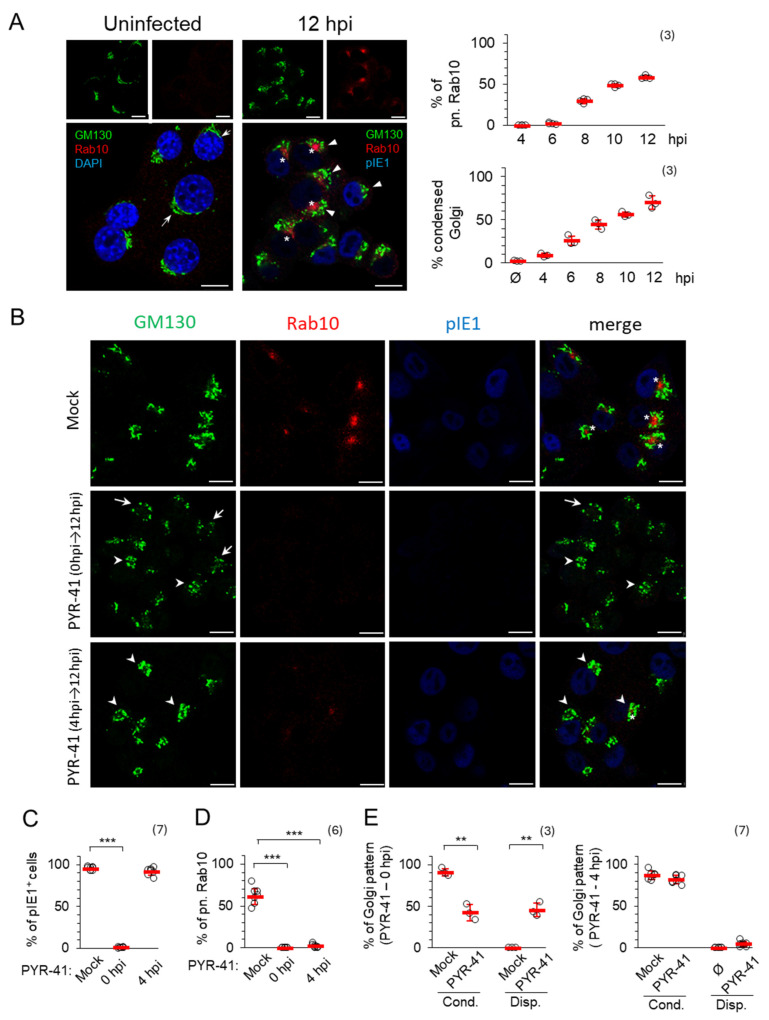
PYR-41 inhibits the formation of pre-AC. (**A**) NIH3T3 cells were infected with ΔFcR MCMV and analyzed for the expression of GM130 (green), Rab10 (red) and pIE1 (blue) by immunofluorescence after 12 hpi. In the uninfected control, the cell nuclei were stained with DAPI (blue). Arrows indicate extended Golgi, arrowheads indicate condensed Golgi, and asterisks indicate perinuclear Rab10 in pre-AC. The percentage of cells expressing condensed Rab10 and GM130 in pre-AC is shown on the right. Means ± SD (red bars) and individual values (empty circles) are shown. The complete experiment is shown in Figure S8A. (**B**) ΔFcR MCMV-infected cells were treated with 15 μM PYR-41 at 0 and 4 hpi or mock treated. After 12 hpi, the expression of GM130 (green), Rab10 (red) and pIE1 (blue) was analyzed. Arrowheads indicate condensed Golgi and arrows indicate dispersed Golgi. Asterisks indicate Rab10 in pre-AC. (**C**–**E**) The ratio of pIE1-positive cells (**C**), cells with perinuclear Rab10 (**D**) and condensed/dispersed Golgi patterns (**E**) in PYR-41-treated and mock-treated cells. Shown are the mean ± SD (red bars) and individual values (empty circles). The number of independent experiments is indicated in parenthesis. Statistical significance was determined using a two-tailed paired Student *t*-test (*** *p* < 0.001; ** *p* < 0.01). Bars—10 μm.

In our previous studies, we have shown that the reorganization of the membrane system and the establishment of the pre-AC are initiated in the E phase of infection [15,16,17,18,19]. In Balb3T3 cells, the basic configuration of the pre-AC, which includes dislocated Golgi (outer pre-AC) and expanded EE-RE/ERC-TGN (inner pre-AC), is established in the majority of cells 5–6 hpi when the cells are infected with an MOI of 10 [15,19]. In NIH3T3 cells, this process is somewhat slower, and the basic configuration was established in 70% of the cells in the 8–12 hpi period (Figure 4A). In these cells, Golgi reorganization into a ring-like configuration was initiated at 4 hpi, as shown by the visualization of the Golgi marker GM130 in the outer pre-AC, while expansion of Rab10 TMs was initiated in the inner pre-AC at 6–7 hpi (Figure 4A and Figure S8A). The observed difference between Balb3T3 and NIH3T3 cells was not related to the type of virus used, as the same kinetics were observed after infection with a recombinant virus lacking the m138 gene (ΔFcR MCMV) (Figure 4A, right) and the wild-type virus (Figure S8B). The pattern of establishment of the basic configuration in NIH3T3 cells is consistent with previously published detailed analyses [15,18,19] and with the processes observed in HCMV-infected cells, where the entire process is much slower and takes two days [12,22,23].

When ubiquitination was inhibited at the time of infection with 15 μM PYR-41 (0 hpi), no nuclear staining of pIE1 was observed (Figure 4B,C), the Golgi structure and location were altered (Figure 4B,E), without expansion of Rab10 TMs (Figure 4B,D), suggesting that PYR-41 prevents pre-AC formation, consistent with the observation that PYR-41 at 0 hpi blocks infection and prevents viral gene expression. The GM130-positive compartments were found either to be unlinked (fragmented) and condensed in perinuclear areas in half of the cells (42.57 ± 9.75%) (Figure 4B, arrowheads) or scattered in small vesicles (45.7 ± 8.47%) in the other half of the cells (Figure 4B, arrows). However, a similar distribution pattern of GM130-positive compartments observed in PYR-41-treated and MCMV-infected cells was also seen in PYR-41-treated and uninfected cells (Figure S9). After 8 h of PYR-41 treatment, the Golgi in these cells was unlinked and condensed, whereas after 12 h, it was dispersed, suggesting the temporal dynamics of Golgi fragmentation in the absence of ubiquitination, which can also be observed in MCMV-infected cells as two subsets of changes. These data are consistent with several observations suggesting that the ubiquitination of Golgi resident proteins serves as a regulatory mechanism to maintain Golgi integrity [50,51] by finely balancing the ubiquitination of Golgi regulatory proteins and either their degradation or a non-degradative post-translational modification to regulate protein interactions for membrane trafficking, Golgi membrane clustering and positioning [51,52,53]. Therefore, Golgi fragmentation and condensation cannot be used as a reliable indicator for the formation of pre-AC in PYR-41-treated MCMV-infected cells. Nevertheless, the experiments with PYR-41 in MCMV-infected and non-infected cells show that Golgi unlinking, although occurring earlier, does not initiate and drive the expansion of Rab10 TMs within the inner pre-AC.

When ubiquitination was inhibited at 4 hpi, the Golgi was condensed (Figure 4B,E) but no perinuclear accumulation of Rab10 was observed (Figure 4B,D) (only 2.1 ± 2.09% in PYR-41-treated and MCMV infected cells in comparison to 61.19 ± 9.27% in control cells), although almost all cells were infected, as they expressed pIE1 (Figure 4B,C). To confirm that PYR-41 did not influence Rab10 dynamics, we tested its expression on uninfected and PYR-41-treated cells, and we found no difference in comparison between uninfected and mock-treated cells (Figure S9). Therefore, the lack of accumulation of Rab10 TMs can be accepted as a reliable result that indicates the absence of pre-AC development in PYR-41-treated cells.

When PYR-41 was added 4 hpi, the GM130-positive membranes were found condensed at 12 hpi, in a ratio similar to that observed after 8 hrs of treatment of uninfected cells (Figure S9) and in mock-treated MCMV-infected cells (81.8 *±* 5.1% in PYR-41-treated and MCMV infected cells in comparison to 86.9 *±* 5.14% in control cells) (Figure 4E). Despite the Golgi reorganization, which may be initiated by infection or by PYR-41 treatment, no expansion of Rab10-decorated membrane compartments was observed at 12 hpi (Figure 4B,D), indicating that ubiquitination-dependent processes are required for the establishment of the inner pre-AC composition.

#### 3.2.2. Ubiquitination Is Required for Integrity of Pre-AC and AC

**Figure 5:**
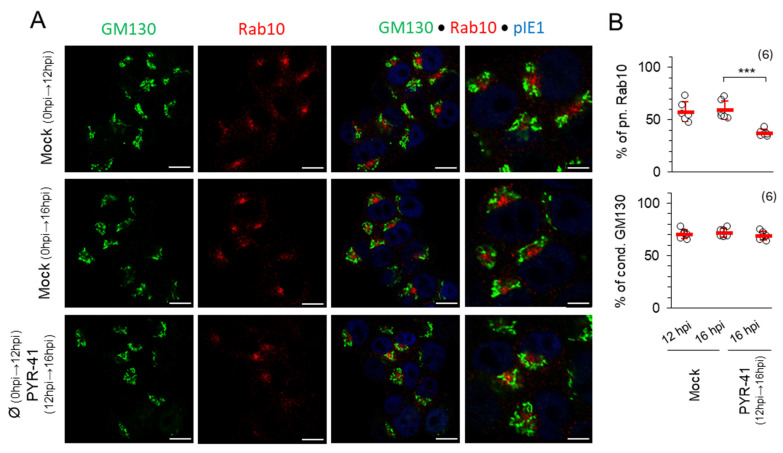
PYR-41 impairs the previously formatted pre-AC. (**A**) NIH 3T3 cells were infected with ΔFcR MCMV and treated with 15 μM PYR-41 at 12 hpi or mock-treated. After 16 hpi, the expression of GM130 (green), Rab10 (red) and pIE1 (blue) was analyzed by immunofluorescence. Bars—10 μm. (**B**) The ratio of cells with perinuclear Rab10 and GM130 in PYR-41-treated and mock-treated cells. Shown are the mean ± SD (red bars) and individual values (empty circles) from six independent experiments. Statistical significance was determined using a two-tailed paired Student *t*-test (*** *p* < 0.001).

Next, we investigated whether ubiquitination is required for the integrity of the previously formed pre-ACs, especially the inner pre-ACs, which consist of numerous extended tubules whose formation and maintenance can be controlled by the ubiquitination of regulatory proteins [54,55,56]. Therefore, we treated the cells with 15 μM PYR-41 at 12 hpi when the pre-AC is fully established and monitored the pre-AC by immunofluorescence until 16 hpi, before viral DNA replication, expression of L genes and maturation of the pre-AC into the AC began (Figure 5). No significant change in Golgi configuration was observed during this period (Figure 5A,B), suggesting that ubiquitination is not required for the maintenance of the outer pre-AC. In contrast, the percentage of cells that developed perinuclear accumulation of Rab10 TMs was similar in mock-treated cells at 12 hpi (57.6 ± 9.3%) and at 16 hpi (59.3 ± 8.9%), whereas the percentage decreased significantly when PYR-41 was present from 12 to 16 hpi (37.2 ± 3.4%). In some cells, the fading of the Rab10 signal was also notable (Figure 5A; PYR-41 12–16 hpi), indicating the disintegration of the inner pre-AC and the detachment of Rab10 from the membranes of the inner pre-AC. These observations suggest that ubiquitination is required for the maintenance of the tubular pattern of the pre-AC.

**Figure 6:**
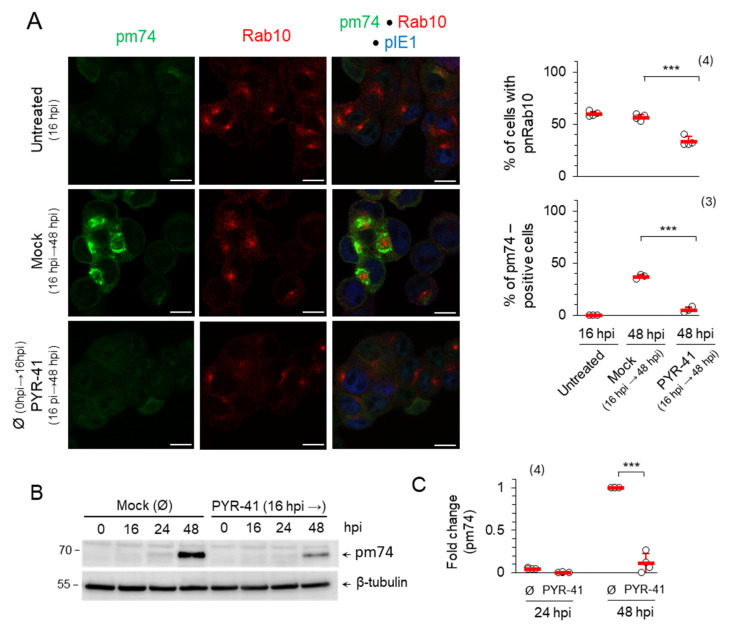
PYR-41 disrupts the AC. (**A**) ΔFcR MCMV-infected cells were treated with 15 μM PYR-41 at 16 hpi or mock-treated. After 48 hpi, the expression of pm74 (green), Rab10 (red) and pIE1 (blue) was analyzed by immunofluorescence. The percentage of cells expressing pm74 and perinuclear Rab10 accumulation are shown on the right as mean ± SD (red bars) and individual values (empty circles). Statistical significance was determined using a two-tailed paired Student *t*-test (*** *p* < 0.001). Bars—10 μm. (**B**) Wt MCMV-infected cells were treated at 16 hpi with 15 μM PYR-41 or mock-treated. At the indicated time points, pm74 expression was determined by Western blot. β-tubulin was used as a loading control. (**C**) Signals were analyzed by ImageJ and normalized to the loading control. Fold changes represent the signal expression relative to the band with the highest intensity in the mock-treated samples. Shown are the mean values ± SD (red bars) and the individual values (empty circles). The number of independent experiments is indicated in parenthesis. Statistical significance was determined using a two-tailed paired Student *t*-test (*** *p* < 0.001).

To investigate whether PYR-41 affects the integrity and formation of the mature AC, it was added at the beginning of the L phase of MCMV infection (16 hpi). The inner AC was marked by Rab10, while the mature outer AC was monitored by the expression of the late envelope protein pM74, as previously described [15,17]. As expected, at the beginning of the experiment (16 hpi) Rab10 TMs accumulated in the inner pre-AC (59.28 ± 2.32% of cells), while pM74 was not yet synthesized (Figure 6A). As the infection progressed (48 hpi), the mature AC was finally established by the accumulation of pM74 in the outer AC of 36.85 ± 2.2% of cells surrounding Rab10 TM accumulation of the inner AC of 56.6 ± 2.9% of cells (Figure 6A). However, treatment with PYR-41 prevented the expression of pM74, and pM74-positive membranes were found in only 5.01 ± 3.0% of cells (Figure 6A), while the accumulated Rab10 TMs in the inner AC were disassembled, resulting in 33.3 ± 4.7% of cells with Rab10 TMs accumulation (Figure 6A). The decrease in pm74 synthesis after the addition of PYR-41 by 16 hpi was detected by Western blot analysis, which confirmed the immunofluorescence data (Figure 6B,C). Therefore, functional ubiquitination in the infected cell is required for the proper synthesis of late MCMV proteins, even when pre-AC is established suggesting that ubiquitination is involved in the regulation of MCMV late gene expression and in the maintenance of AC.

#### 3.2.3. Ubiquitination in the Late Phase of Infection Is Required for the Production of Infectious MCMV Virions

**Figure 7:**
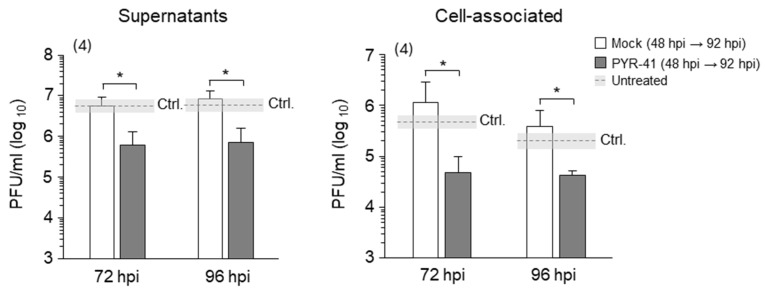
Treatment with PYR-41 in the late phase of infection inhibits the production of infectious virions. NIH3T3 cells were infected with wt MCMV. After 48 hpi, the cell culture medium was replaced with fresh medium, and cells were treated with 15 μM PYR-41 or mock-treated. The supernatants or cell lysates were harvested after 72 and 96 hpi and infectious virion production was determined using the plaque assay. The mean values of four independent experiments are plotted; the error bars show the standard deviation. Statistical significance was determined using the Mann–Whitney test (* *p* < 0.05). Ctrl.—control level of virus production in mock-treated cells without changing the cell culture medium 48 hpi.

We have shown that PYR-41 treatment inhibits virion production in cells when added prior to pre-AC formation (Figure 3F). Since the results presented here suggest that functional ubiquitination is important, at least in part, for maintaining the integrity of the established AC (Figure 6), we tested whether virion production in MCMV-infected cells is impaired after the addition of PYR-41 at 48 hpi. To minimize the influence of previously synthesized virions during the experimental period, the old cell culture media were replaced with fresh media after 48 hpi, followed by the addition of 15 μM PYR-41 and quantification of released (supernatant) and cell-associated (cell lysates) infectious particles after 72 and 96 hpi using the plaque assay. In line with the results shown in Figure 6, treatment with PYR-41 reduced the amount of cell-associated and released infectious particles to 10% of the control value (Figure 7), suggesting that functional ubiquitination is still required for virion production even when the AC is fully established and late proteins are synthesized. Although these data suggest that PYR-41 impairs secondary envelopment events, the reduction in components required for the construction of infectious particles (i.e., the synthesis of late proteins) cannot be excluded.

#### 3.2.4. Ubiquitination Contributes to the Process of Extensive Tubulation of Rab10-Positive Membranes Within the AC

Several lines of evidence suggest that CMV envelopment occurs at membranes derived from endosomal compartments [24,57,58,59,60,61] that are extensively expanded in the AC of HCMV- and MCMV-infected cells [15,22,62]. Our recently published results show the expansion of Rab10 TMs arising in the SNX27:Retromer:ESCPE-1-driven EE domains of MCMV-infected cells [17,18]. Among several different tubular domains that are expanded within the AC [15], the expansion of Rab10 TMs is a useful early marker for AC biogenesis [18]. This tubulation is highly dynamic and is maintained within the fully formed AC in the L-phase of infection, as demonstrated by long-term live cell imaging of MCMV-infected NIH3T3 EGFP-Rab10 cells using fluorescence-enhanced digital holotomographic microscopy (DHTM) [18].

**Figure 8:**
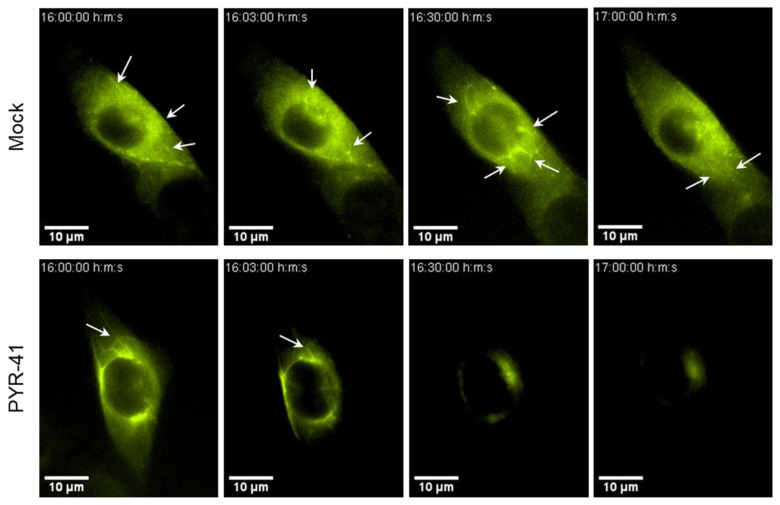
PYR-41 disrupts Rab10-associated tubulation in the established AC. NIH3T3 EGFP-Rab10 cells were infected with wt MCMV. At 16 hpi, PYR-41 was injected into the tissue culture medium (15 μM concentration), and cells were imaged live with fluorescence-enhanced DHTM for 1 h. Screenshots at the indicated time points are shown and a full time-lapse in Video S1 (Mock) and Video S2 (PYR-41). The arrows indicate expanded Rab10-EGFP-positive tubules in AC.

Since we observed PYR-41-induced disassembly of Rab10 TMs within the AC (Figure 5 and Figure 6), we next investigated whether functional ubiquitination is required for Rab10-associated tubulation by live imaging of MCMV-infected NIH3T3 EGFP-Rab10 cells [18]. In these cells, EGFP-Rab10 synthesis was induced by doxycycline for 48 h. The expressed EGFP-Rab10 is mainly distributed in the cytosol and recruits only a few membranes in the juxtanuclear region, resulting in a weak fluorescence signal, whereas EGFP-Rab10 accumulates at membranes of TREs after infection [18]. At 16 hpi, EGFP-Rab10 was found concentrated in the perinuclear region representing the AC, with dynamically expanding tubules emanating from the edges of the accumulation (Figure 8; Videos S1 and S2). However, almost immediately after the addition of 15 μM PYR-41 (3 min), these EGFP-Rab10-positive tubular structures collapsed. Moreover, the overall fluorescence signal gradually faded and was almost completely extinguished after about 1 h of treatment. This result is consistent with our previous observations indicating that the lack of functional ubiquitination affects the integrity of AC (Figure 6). However, the rapid silencing effect of PYR-41 on the elongated AC tubules suggests that its influence on the tubular part of the AC structure leads to the final disintegration of AC in some cells (Figure 8).

#### 3.2.5. WASHC1 Is Hyperubiquitinated in MCMV-Infected Cells, but Its Presence Is Dispensable for the Accumulation of Rab10 TMs in Pre-AC and Virion Assembly

**Figure 9:**
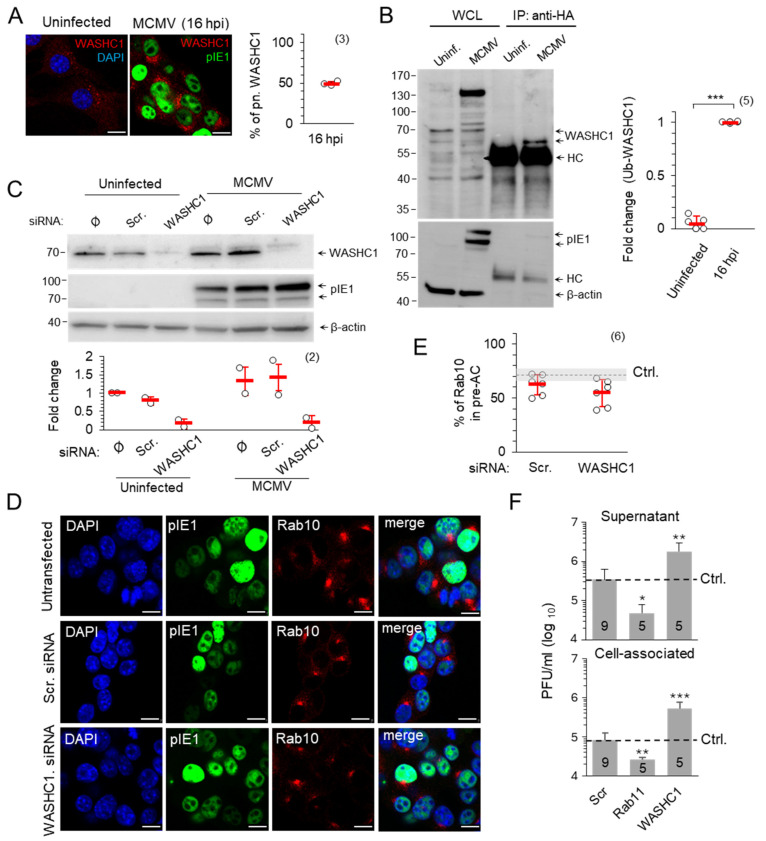
WASHC1 in MCMV-infected cells. (**A**) NIH 3T3 cells were infected with wt MCMV or left uninfected and analysed 16 h later by immunofluorescence for the expression of pIE1 (green) and WASH1C (red). DAPI (blue) was used to stain the cell nuclei. The percentage of cells with perinuclear WASHC1 (mean ± SD (red bar)) is shown on the right. (**B**) Doxycycline-treated NIH3T3 HA-Ub cells were infected with wt MCMV or left uninfected. After 16 h, 10% of cells were lysed for WCL and 90% of cells were lysed for the immunoprecipitation of ubiquitinated proteins with rabbit anti-HA. The expression of WASHC1, pIE1 and β-actin was visualized with the corresponding primary and secondary antibodies. The fold change represents the signal expression of Ub-WASHC1 relative to the uninfected cells. HC—Heavy chain of anti-Rb IgG pAb. (**C**,**D**) NIH3T3 cells were transfected with Scr. or WASHC1 siRNA or left untransfected (control). After 48 h, cells were infected with wt MCMV for 16 h or left uninfected and analyzed either by Western blot (**C**) or immunofluorescence (**D**). Signals were normalized to β-actin and fold change represents signal expression relative to uninfected and mock-treated samples. (**D**) Triple staining of Rab10 (red), pIE1 (green) and DAPI (blue) was analyzed by confocal imaging. (**E**) Percentage of MCMV-infected (pIE1-positive) cells with perinuclear accumulation of Rab10 in Scr. and WASH1 siRNA-treated cells at 16 h post-infection, shown as mean ± SD. Ctrl., control the level in non-transfected cells. The number of independent experiments is indicated in parenthesis. (**F**) Cells were treated with Scr., Rab11 or WASHC1 siRNA for 48 h and infected with wt MCMV. Supernatants and cell lysates were harvested at 48 hpi, and the number of infectious units was determined by plaque assay. The number of experiments is indicated in the bars. The error bars show the standard deviation. Statistical significance was determined using the Mann–Whitney test (* *p* < 0.05, ** *p* < 0.01, *** *p* < 0.001). Bars—10 μm.

Tubulation at endosomes is regulated by proteinaceous coats that serve as molecular scaffolds for the induction of a tubular shape [63,64]. These coats are controlled by the Sorting Nexin (SNX) family of proteins, which are recruited to recycling endosomal domains upon cargo and phosphatidylinositol-3-phosphate (PI(3)P) recognition [63,65]. We have shown that the SNX27:Retromer:ESCPE-1 coating complex activates Rab10-tagged tubulation in pre-AC [17]. The generated tubules are rapidly detached from the endosomal surface, and one of the best elucidated mechanisms involves proteins of the Wiskott–Aldrich syndrome protein and SCAR homologue (WASH) complex that activates actin polymerization [54,56,64]. Actin microfilaments then trigger membrane tension at the root of the tubule, which contributes to carrier fission with the help of proteins such as dynamin and PROPPINs [64]. Considering that FAM21 from the WASH complex can bind and recruit Vps35 from retromer, bridging the gap between the SNX27:Retromer:ESCPE-1 coating complex and the WASH complex [64,66], we investigated the role of WASHC1 in the formation of the AC (Figure 9).

The membrane association of WASHC1 increased in the AC (Figure 9A), which is expected as WASHC1, part of the pentameric WASH multiprotein complex, can interact with the Vps35 components of retromers [64,66,67] that also accumulate in the AC [17]. The activation of WASHC1 is regulated by K63 ubiquitination which induces its active conformation, enabling the carboxy-terminal VCA (veroprolin homologous or WH2, central hydrophobic, and acidic) motif to bind actin and Arp2/3, stimulating actin filament nucleation and preceding tubule fission [56]. However, WASH*C*1 activation follows the principle of rheostat, where deubiquitinase USP7 prevents both WASH*C*1 hyperubiquitination and hypoubiquitination to maintain the optimal endosomal actin level on the root of the extending tubule [54]. The immunoprecipitation of proteins on NIH3T3 HA-Ub cells showed a highly increased level of ubiquitinated WASHC1 in MCMV infected cells compared to uninfected cells (the fold changes in uninfected cells were only 0.05 ± 0.06 in comparison with 1.0 ± 0.0 in MCMV infected cells) (Figure 9B). Although increased WASHC1 ubiquitination could serve as a signal for degradation, we did not observe the decrease in WASHC1 expression in infected cells neither by immunofluorescence (Figure 9A) nor by Western blot analysis (Figure 9C). The depletion of WASHC1 by siRNA, which resulted in an almost complete loss of WASHC1 from uninfected and MCMV-infected cells (Figure 9C), did not affect the perinuclear accumulation of Rab10 TMs (Figure 9D,E), indicating that WASHC1 is dispensable for the accumulation of recycling EE/RE membranes. Therefore, we concluded that MCMV affects the WASH*C*1 function by hyperubiquitination rather that stimulating its degradation.

Although the WASH complex can be recruited by both the retromer and SNX27 [68,69,70], two components shown to be required for Rab10-dependent tubulation [17,18], its function does not contribute to the process of tubule initiation since WASHC1 siRNA-treated cells developed perinuclear accumulation of Rab10 TMs to a similar extent as Scr-treated cells (Figure 9D,E). However, the WASH-associated tubule fission function [71] may contribute as a restriction factor for tubule growth within the AC, as several studies have shown that the depletion or hyperubiquitination of WASHC1 leads to abundant tubulation and impairs cargo recycling [54,56,67].

The WASH-facilitated tubulation in the MCMV-infected cell could be related to the envelopment of the tegumented MCMV capsids by a finger-like mechanism that has been described for apoptosis [72]. Therefore, we hypothesized that the number of virions produced would be proportional to the amount of available tubular extensions and that factors which promote tubule growth would increase, while factors which restrict tubule growth would decrease virion production. To test this hypothesis, cells with siRNA-depleted WASHC1 and Rab11 were infected and the amount of produced and released intracellular infectious units was determined (Figure 9F). The reference level of infectious unit production was determined in untransfected cells (Figure 9F, dashed line). Depletion of Rab11, a factor required for the maintenance of the tubular recycling system of the ERC, decreased infectious unit production nearly tenfold, while the depletion of WASHC1 increased virion production nearly fivefold (Figure 9F). The lack of difference between cell-associated and released infectious units (Figure 9F) suggests a change in the envelopment process rather than the release of infectious units. Taken together, these data suggest that the ubiquitination-associated rheostatic function of WASH may control the final assembly of infectious CMV virions.

## 4. Discussion

In this study, we investigated the role of ubiquitination in the control of CMV infection and cytoplasmic assembly. We used PYR-41, a small chemical inhibitor that rapidly abolishes ubiquitination by acting on the ubiquitin-activating enzyme E1 [37,38,39]. Inhibition of ubiquitination prior to infection completely prevented the development of infection even when high MOI was applied (Figure 1). Therefore, we titrated PYR-41 to a concentration that allows long-term treatment at various stages after infection without driving the cells into apoptosis. We showed that ubiquitination is involved in the reorganization of the cell’s membrane system, in the progression of the replication cycle, in the expression of late genes and in the production of infectious viruses. We have particularly focused on the reorganization of the membrane system, which may be important for secondary envelopment, a process of the membrane wrapping of cytoplasmic clusters of viral capsids embedded in the tegument matrix. By monitoring the accumulation by Rab10 TMs within the AC, a previously established SNX27:Retromer:ESCPE-1-dependent process [17,18], we showed that the ubiquitination is required for the reorganization of the membrane system (Figure 4). Ubiquitination is required for both the maintenance and expansion of Rab10 TMs, as confirmed by confocal microscopy (Figure 5 and Figure 6) and long-term live cell imaging (Figure 7). A ubiquitin-dependent mechanism controlling this expansion may rely on the recruitment of WASH complexes to the Vps35 component of retromer within the SNX27:Retromer ESCPE-1 complex [68,69], which may direct tubular growth and fission through ubiquitin-dependent rheostatic control [54]. When properly ubiquitinated, WASHC1 facilitates the termination of tubular growth, whereas when hyperubiquitinated, as observed in MCMV-infected cells, tubular growth is enhanced and allows the elongation of Rab10 TMs due to inefficient fission. While it is known that the generation of endosomal tubules can occur independently of functional WASHC1, both WASHC1 and actin are necessary for tubular detachment [64,71]. The overproduction of excessively elongated tubules may be related to the development of a sufficient number of tubular membranes for viral envelopment, and the depletion of WASHC1 by siRNA would therefore facilitate the production of more infectious virions (Figure 9F).

Ubiquitination is a pleiotropic function of the cell that is used to regulate many cellular processes. Therefore, it is not surprising that CMV could use the ubiquitin pathway to manipulate the proteome of the infected cell in favour of viral replication [31]. The mammalian genome contains more than 600 genes encoding ubiquitin ligases [73], which produce more than 650 Ub-conjugated proteins as found in HeLa cells [74], and the expression of more than 80% of them is controlled by their degradation at proteasomes [75]. Many cellular proteins are also regulated by ubiquitination, as has been shown for the rheostatic function of WASH complexes [54]. CMV can also utilize the ubiquitin system to regulate the number of viral proteins, their stability or even their function. Therefore, it is not surprising that the blockade of proteasomal functions impairs cellular processes and prevents the replication of MCMV, as it has been described that inhibitors of degradation and ubiquitination affect the replication cycle for HCMV [34,35,36] and other viruses [76,77].

We used PYR-41, a well-established inhibitor of the ubiquitin-activating enzyme (E1) [37], which is not only used to elucidate basic ubiquitin-dependent cellular processes but is also being considered as a therapeutic agent in various malignancies because it stimulates apoptosis and cross-links signalling kinases such as Bcr-Abl and Jak2 [37,78,79]. Although we applied the inhibitor at a concentration that did not affect cell viability and thus avoided its cytotoxic effect, other off-target effects of PYR-41 cannot be neglected, such as the attenuation of the NFκB factor, inhibition of p53 degradation and cellular stress response [37,78]. Interestingly, some authors point out the importance of NFκB for IE expression and L gene expression [80,81]. Therefore, we plan to extend our further investigations with other methods, such as specific siRNA silencing or CRISPR tools.

In this study, we investigated the effect of PYR-41 on the establishment of the infection by monitoring GFP expression, which is controlled by IE gene promoters [41], and the expression of pIE1, an abundant IE protein (Figure 1). It was previously observed that proteasome inhibitor MG132 prevented pIE1 and pIE2 synthesis in HCMV-infected cells when they were infected with a low MOI (0.01–0.5), but not with high MOI (5–10) [35,36]. However, in our experiments, the synthesis of pIE1 was prevented even when the cells were infected with a high MOI (10) and this discrepancy is probably related to the different inhibitors used. MG132 inhibits degradation due to a blockade of the ubiquitin-proteasome system [82], while PYR-41 might show an effect on a broader range of proteins, considering that the effect of ubiquitination is not limited to proteasomes [54,56,83,84]. Nevertheless, the broad effect of PYR-41 on E and L gene expression is comparable to observations in HCMV-infected cells treated with proteasomal inhibitors (MG132 or lactacystin) [35,36] or inhibitor of the Cullin-Rock ubiquitin ligase (MLN4924) [31].

Ubiquitination is required for the expression of the first wave of MCMV regulatory genes in the IE and E phase of infection, and ubiquitination is used to regulate the decay or function of these proteins, as we have found by ubiquitination of pIE1 (Figure 1 and Figure 2). We found that a concentration of 15 μM PYR-41 can be applied after 4 hpi to monitor the progression of the replication cycle in the long term and to study the effect of Ub on the establishment and maintenance of a large cytoplasmic cluster of membranous organelles known as the AC.

To investigate the effect of PYR-41 on the establishment of the AC, we focused on the earliest signs of reorganization of the membrane system into the AC that can be monitored by immunofluorescence analysis. In our previous studies, we have established in detail that the basic configuration of the AC, which includes the unlinking, dislocation and reorientation of the entire Golgi into a perinuclear ring-like configuration, envelops the expanded early endosomal compartments, ERC and TGN into a new organelle structure [15,17]. In MCMV-infected cells, the basic configuration is rapidly established 6–7 hpi in Balb3T3 cells [15], and the entire structure, termed pre-AC during the E phase of infection until viral DNA is replicated and the L genes are expressed, develops during E phase. The earliest event that can be observed by immunofluorescence is the unlinking and relocation of the Golgi using various Golgi markers, followed by the enlargement and expansion of the EE, RE/ERC and TGN membrane domains and many domains at intermediates between these compartments [15]. This expansion is associated with increased membrane recruitment of many host cell proteins, particularly proteins involved in or regulating membrane tubulation processes [15,17,18]. We observed the over-recruitment of Rab10 as the earliest marker that provides the best resolution for identifying the earliest events and as a marker of well-characterized endosomal tubulation, which is a hallmark of pre-AC [15,17,18]. Unfortunately, we could not use Golgi rearrangement to monitor the effect of PYR-41 on the establishment of pre-AC because PYR-41 itself restructures the Golgi in a multi-hour sequence characterized first by Golgi unlinking and compaction and later by Golgi fragmentation and dispersal (Figure S9). This observation is consistent with several reports demonstrating the role of non-proteolytic ubiquitination of Golgi proteins in maintaining the Golgi integrity in uninfected cells [50,51,52]. Therefore, we mainly focused on the expansion of the Rab10-positive domain, which is well characterized and may represent the critical point for understanding the process of how MCMV acquires the envelope.

The expansion of the Rab10-TM, an essential component in the development of tubular recycling endosomes [85,86], is associated with tubulation within the AC [17,18]. These tubular extensions are highly dynamic and are initiated in the pre-AC and maintained in the AC [18]. They are generated by a cascade recruitment of SNX27:Retromer:ESCPE1 complexes [17] in the SNX3-dependent zone of EEs [87]. These complexes recruit cargo proteins with appropriate short linear motifs (SLIMs) and segregate them into an EE domain for recycling to the PM [63]. SNXs with BAR domain within ESCPE-1 complexes (SNX1/2 and SNX5/6) generate membrane curvature to initiate tubulation [64,88] and can recruit phosphoinositide kinase to change the lipid composition from PI(3)P to PI(4,5)P2 [89], which directly recruits EHBP1 [90], followed by the recruitment of activated Rab10 and its effectors [90], including motor proteins that extend the membrane along microtubules and generate tubular extensions [85]. The fission of these extensions generates endocytic carriers by the multi-step recruitment of effector proteins. In the first step, both SNX27 and Vps35, components of the retromer complex, recruit WASH complexes [68,69,70], which activate actin polymerisation near the membrane [64,91] and initiate the elongation and constriction of the tubule [64,71]. This narrowing is required for the next step, which involves the recruitment of proteins at the root of the tubule, that mediate scission, such as dynamin or PROPPINs, and terminate the development of tubular extensions [56,92]. The dynamics of WASH complexes at endosomes is regulated by the TRIM27 ubiquitin ligase, which is recruited by retromer and facilitates K63-linked ubiquitination of the WASHC1 component resulting in its release from an auto-inhibited state [91]. Ubiquitination of WASHC1 also acts as a rheostat that controls the intensity of tubular growth [54]. Too little ubiquitination or too much ubiquitination may inhibit the activity of WASH, resulting in the prolonged growth of tubular extensions [54]. In addition to WASH, the tubule growth may be regulated later at the terminal scission step by a Rab10-dependent [93] recruitment of the MICAL-L1, PACSIN2 and EHD1 complexes [86]. These cleavage mechanisms are very dynamic in uninfected cells, resulting in a short residence time of Rab10 at the membranes [94]. In MCMV-infected cells, both scission mechanisms are likely tuned to promote tubular growth.

In this study, we have provided evidence for the hyperubiquitination of WASHC1 in MCMV-infected cells indicating a shift in the rheostat mechanism towards dysregulation in the WASHC1 function (Figure 9). Considering that tubular growth can proceed even without WASH but not its fission [64,71,92], the outcome is expected to be the promotion of the tubule growth. In addition to promoting tubule growth within the AC, tubule termination is apparently impaired in CMV-infected cells, resulting in continuous tubule elongation [18] and inhibited endosomal recycling [16,95,96]. However, at this stage we do not yet fully understand the mechanism of tubule fission and further experiments are required to determine what is impaired in MCMV-infected cells. Nonetheless, facilitated tubular growth can be beneficial for the needs of CMV in the envelopment process.

The mechanism of secondary envelopment and the organelle source for membrane envelopment of tegumented virions are largely unknown, and many mechanistic details of this process are unclear. Electron microscopic studies suggest both the budding of tegumented capsids into multivesicular bodies and membrane wrapping around cytoplasmic tegument condensate containing capsids [12,97,98,99,100]. In MCMV-infected cells, the physical constraints that must be solved in this process are further complicated by the fact that the envelopment includes large structures with multiple capsids [97]. In addition to the physical constrains, the sorting and concentration of the various viral glycoproteins at the envelopment membrane is also a critical issue. Since the viral glycoproteins should reach the envelopment membrane through membrane flux [61,101,102,103,104], their sorting and concentration should be subject to the host cell mechanisms for cargo sorting. These mechanisms are present in EEs, and several studies suggest that the endocytic process and endosomal membranes are involved in envelopment [59,60,61,102]. Analysis of SLIMs in major viral glycoproteins shows that they can be sorted by the SNX27:Retromer retrieval mechanism [17], suggesting that they are retrieved by the clathrin-independent cargo-sorting pathway, which involves sequestration into Rab10-dependent carriers and their onward transport into the Rab8/Arf6-dependent tubular subset of the ERC [105]. Some glycoproteins, e.g., gH, have a short cytoplasmic tail and can also be sorted by other pathways. Furthermore, there are no known sorting signals for MVBs in viral glycoproteins. In addition to the sorting of viral glycoproteins to tubular recycling membranes, the inhibition of the exit pathway, as demonstrated by inhibited endosomal recycling in CMV-infected cells [16,95,96], could also contribute to the accumulation of viral glycoproteins at these membranes. Therefore, we favour the hypothesis that secondary envelopment occurs through tubular membranes of recycling endosomes that wrap around the tegumented capsid with a finger-like mechanism, as recently described for macroautophagy [72,106].

The finger-like wrapping by tubular membranes originating from recycling endosomes described for macroautophagy could involve tubular membranes from different sources and not from the same organelle [72]. Our previous studies, based on the analysis of protein markers, have shown that the inner AC is formed by the accumulation of tubular membranes from different sources, such as membranes carrying class I and class II Arf proteins, or by the expansion of phosphatidyl-serine-rich membranes of ERC origin [15]. These tubular membranes can be dilated by altering v-ATPase trafficking [107,108] and wrap around tegumented capsids by ESCRT-dependent fusion and dynamin-dependent conversion [72] into bilayered organelles containing single or multicapsid virions. Consistent with this hypothesis, any host cell factor or process that promotes the growth and expansion of tubules and their accumulation within the AC would increase the chance of envelopment and increase virion production, and conversely, the lack of any host cell factor that maintains tubule integrity would decrease virion production. Our preliminary approach by deleting WASHC1 and Rab11 in this study supports this hypothesis. Depletion of WASHC1, which is known to induce the elongation of TREs and reduce the exit of cargo into the recycling route [56,67,69,109], increased virion production almost fivefold, while the depletion of Rab11, the known master Rab protein for maintaining the tubular recycling membranes that form the ERC, decreased virion production for almost tenfold (Figure 9F). Ubiquitination could be an important factor in creating sufficient amounts of tubular membranes for envelopment.

We found increased ubiquitination of WASHC1, which could facilitate endosomal tubulation and AC formation. However, it would be important to determine whether other proteins that regulate recycling tubule formation can also be regulated by ubiquitin. It is known that Rab11 effectors such as EHD1, MICAL-L1, Rab11-FIP1c, Rab11-FIP2 and Rab11-FIP5 are ubiquitinated by RFFL-E3 ligases, and this modification is important for endosomal recycling [55]. Considering that these proteins play an important role in tubulation [110], the membrane modification that precedes AC formation, it is tempting to speculate that ubiquitination of these proteins may also be required for the biogenesis and integrity of ACs. Accordingly, it is reasonable to expect high redundancy in the tubulation machinery, which may explain our observation that WASHC1 contributes to, but is not essential for, Rab10 TM development. Interestingly, the coalescence of ERC in RFFL-DN cells [55] resembles the AC phenotype observed in CMV-infected cells.

The ubiquitin-mediated modulation of cellular and pathogenic proteins has also been observed in infections with other large DNA viruses. Successful infection with herpes simplex virus 1 (HSV-1) is strongly dependent on functional ubiquitin-dependent processes in the host cell [111,112]. Interestingly, HSV-1 encodes Infected Cell Protein 0 (ICP0), the E3 ubiquitin ligase, which promotes infection by modulating the cellular proteome [113] but can also attenuate the host’s anti-inflammatory response [114,115]. Functional ubiquitination was also found to regulate the replication cycle of Kaposi’s sarcoma herpes virus (KSHV) [116], as well as viral gene expression and the anti-inflammatory response in a host infected with vaccinia virus [117]. In addition, in an experimental model using proteasome inhibitors and PYR-41, the authors were also able to show that orthopoxviruses require appropriate ubiquitination for the correct expression of early and late viral proteins [118]. The manipulation of ubiquitination in infected cells is therefore not limited to cytomegaloviruses and regulates the outcome of numerous viral infections.

## 5. Conclusions

Our study shows that the Ub system is intensively used in all phases of the MCMV replication cycle, including the reorganization of the membrane system into AC. From the perspective of the virus, it would make sense to target such a pleiotropic system to comprehensively reorganize the infected cell. Targeting the rheostatic control of membrane tubulation by ubiquitination and expansion of tubular membranes within the AC could be favourable for the creation of a suitable environment for virion envelopment. The coding potential of MCMV is endowed with a gene that can perform such a function. The M48 protein with a deubiquitinating domain, which is embedded in the tegument and essential for virus growth [119], can be used to regulate the extent of ubiquitination on proteins that form the rheostatic control of membrane tubulation within the AC, thereby optimizing the amount of growing tubular membranes for wrapping-based envelopment. Therefore, this study paves the way for further investigations of a complex interaction of viral gene products with the homeostatic control of the organization of the membrane system by the host cell.

## Data Availability

The data presented in this study are available on request from the corresponding authors.

## Supplementary Materials

The following supporting information can be downloaded at: https://www.mdpi.com/article/10.3390/life15081212/s1, Figure S1: Ubiquitination inhibited by PYR-41 prevents infection with MCMV (related to Figure 1A); Figure S2: Immunofluorescence analysis of pIE1 expression in PYR-41-treated MCMV-infected cells (related to Figure 1B); Figure S3: PYR-41 (15 μM) has no effect on the viability of MCMV-infected cells; Figure S4: Complete Western blots related to Figure 1D and Figure 3A,B; Figure S5: NIH3T3 HA-Ub cell lines with inducible expression of HA-Ub constructs; Figure S6: Complete Western blots related to Figure 2; Figure S7: Complete Western blots related to Figure 3; Figure S8: Kinetics of pre-AC formation in MCMV-infected NIH3T3 cells (related to Figure 4A); Figure S9: The effect of PYR-41 on the expression of Rab10 and GM130 in uninfected cells; Figure S10: Complete Western blots related to Figure 6B; Figure S11: Complete Western blots related to Figure 9C; Video S1: Life cell imaging of EGFP-Rab10 in mock-treated MCMV-infected cells (16–17 hpi); Video S2: Life cell imaging of EGFP-Rab10 in PYR-41-treated MCMV infected cells (16–17 hpi).

## Abbreviations

The following abbreviations are used in this manuscript:

AC	Assembly compartment
AF	Alexa Fluor
CMV	Cytomegalovirus
Ctrl	Control
DHTM	Digital Holotomographic Microscopy
E	Early
EE	Early endosomes
ERC	Endosomal recycling compartment
IE	Immediate early
GFP	Green fluorescence protein
HA	Haemagglutinin
HCMV	Human Cytomegalovirus
IP	Immunoprecipitation
L	Late
mAb	Monoclonal antibody
MCMV	Murine Cytomegalovirus
MOI	Multiplicity of infection
pAb	Polyclonal antibody
PFU	Plaque forming unit
POD	Peroxydase
RE	Recycling endosomes
Scr	Scrambled
SNX	Sorting nexin
SD	Standard deviation
TGN	Trans-Golgi network
TRE	Tubular recycling endosomes
Ub	Ubiquitination
WASH	Wiskott–Aldrich syndrome protein and SCAR homologue
WCL	Whole cell lysates
