# Supplementary material for: Ubiquitination Regulates Reorganization of the Membrane System During Cytomegalovirus Infection
Source: Life (Basel). 2025 Jul 31;15(8):1212. doi: 10.3390/life15081212 (PMC12387746; doi:10.3390/life15081212)
Supplement: Supplementary file 1 [file life-15-01212-s001.zip › Radic et al_Supplementary material.pdf]

Article

Ubiquitination Regulates Reorganization of the Membrane System During Cytomegalovirus Infection

Barbara Radić [1,†], Igor Štimac [1,†], Alen Omerović [1], Ivona Viduka [1], Marina Marcelić [1], Gordana Blagojević Zagorac [1,2], Pero Lučin [1,2,*,‡] and Hana Mahmutefendić Lučin [1,2,*,‡]

[1] Department of Physiology, Immunology and Pathophysiology, Faculty of Medicine, University of Rijeka, Braće Branchetta 20, 51000 Rijeka, Croatia; barbara.radic@uniri.hr (B.R.); igor.stimac@uniri.hr (I.Š.); alen.omerovic@uniri.hr (A.O.); ivona.viduka@uniri.hr (I.V.); mmarcelic@uniri.hr (M.M.); gordana.blagojevic@uniri.hr (G.B.Z.)
[2] Campus University Center Varaždin, University North, Jurja Križanića 31b, 42000 Varaždin, Croatia
* Correspondence: pero.lucin@uniri.hr (P.L.); hana.mahmutefendic@uniri.hr (H.M.L.)
† These authors have contributed equally to this work.
‡ These authors have contributed equally to this work and share senior authorship.

1. Supplementary Figures

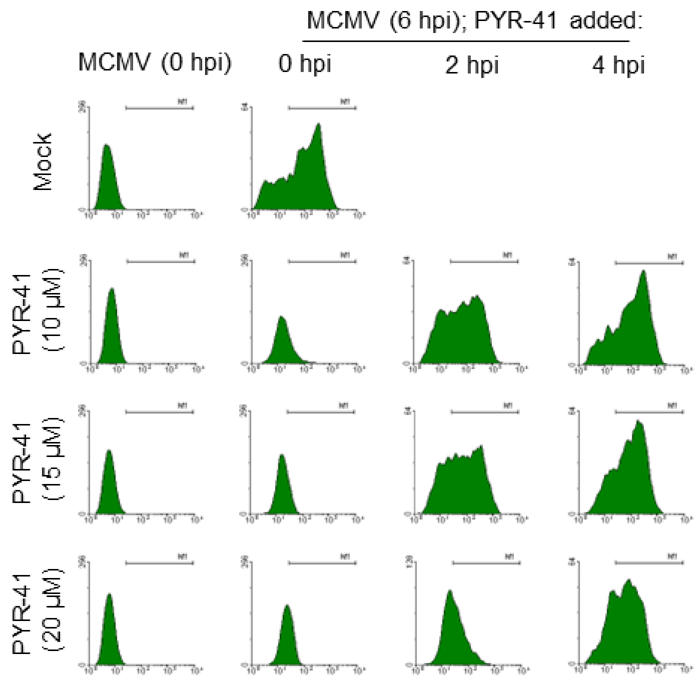

Figure S1. Ubiquitination inhibited by PYR-41 prevents infection with MCMV (*related to Figure 1A*). NIH3T3 fibroblasts were infected with C3X GFP MCMV (MOI of 10) and treated with the indicated concentrations of PYR-41 at the time of infection (together with the virus), 2 hpi or 4 hpi. After 6 hpi. GFP expression was determined by flow cytometry. The results are shown as histogram profiles of the illustrated experiments. M1 indicates GFP-positive cells.

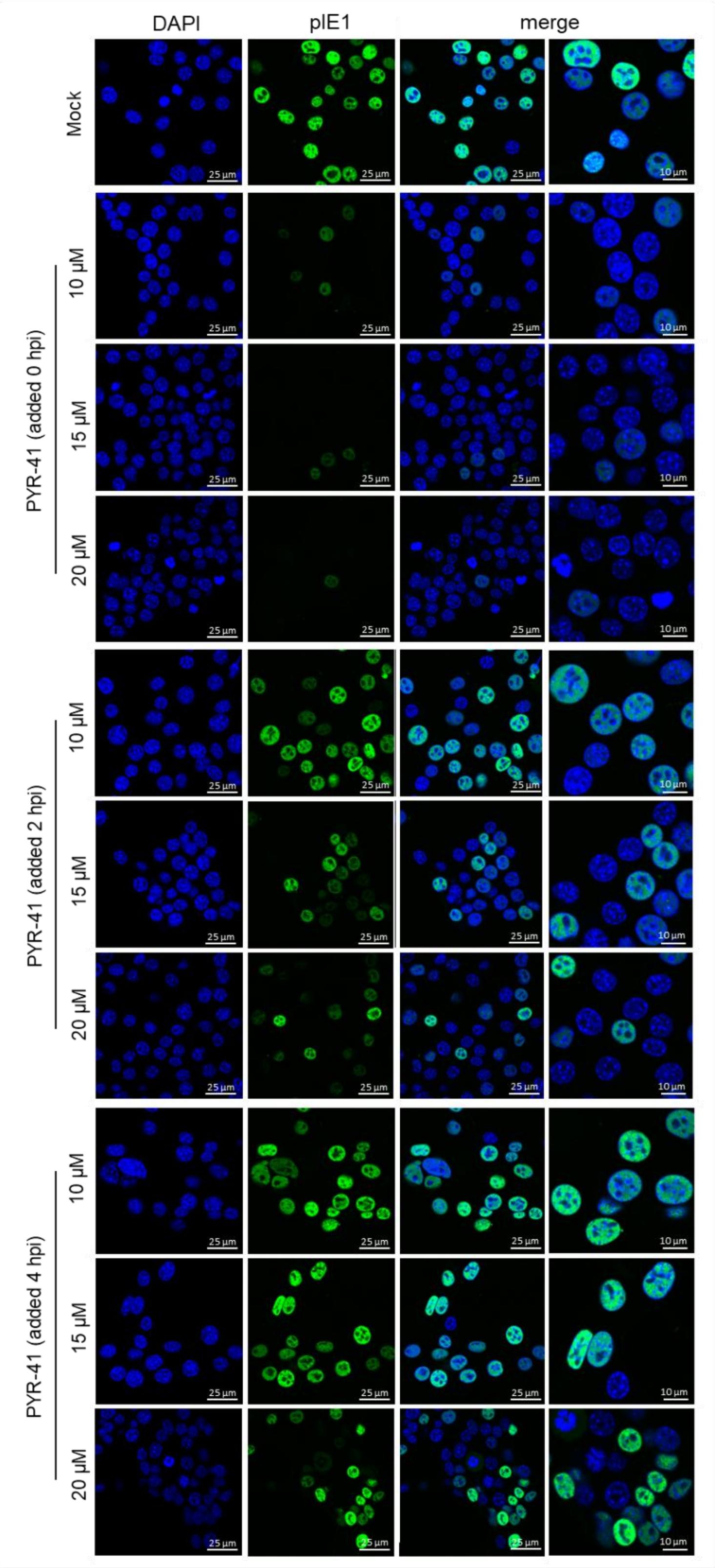

Figure S2. Immunofluorescence analysis of pIE1 expression in PYR-41-treated MCMV-infected cells (*related to Figure 1B*). NIH3T3 cells were treated with the indicated concentrations of PYR-41 and infected with Δ138 MCMV (MOI of 10) for the indicated time periods. After 6 hours p.i., cells were fixed, permeabilized and pIE1 expression (green) together with DAPI (blue) was detected by confocal imaging.

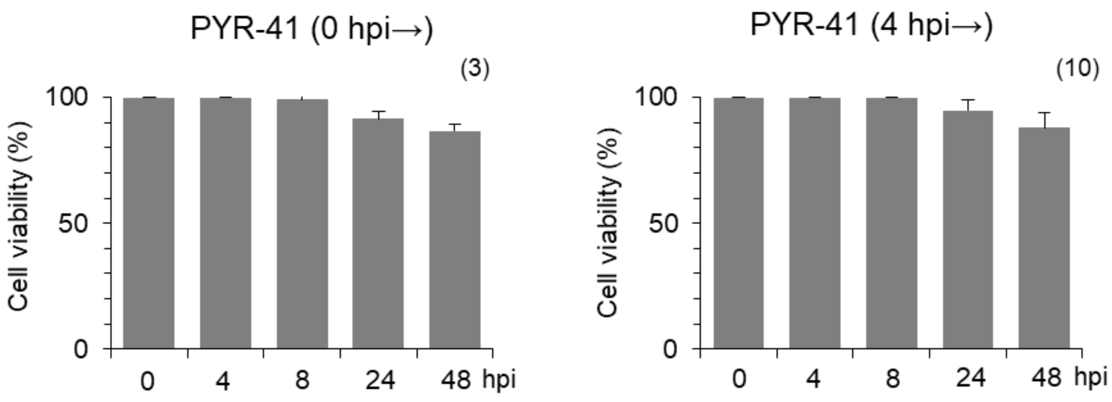

Figure S3. PYR-41 (15 μM) has no effect on the viability of MCMV-infected cells. NIH 3T3 cells were infected with MCMV wt (MOI of 10), and PYR-41 (15 μM) was added at 0 hpi (**A**) or 4 hpi (**B**). Shown are the mean ± SD. The number of independent experiments is indicated in parenthesis.

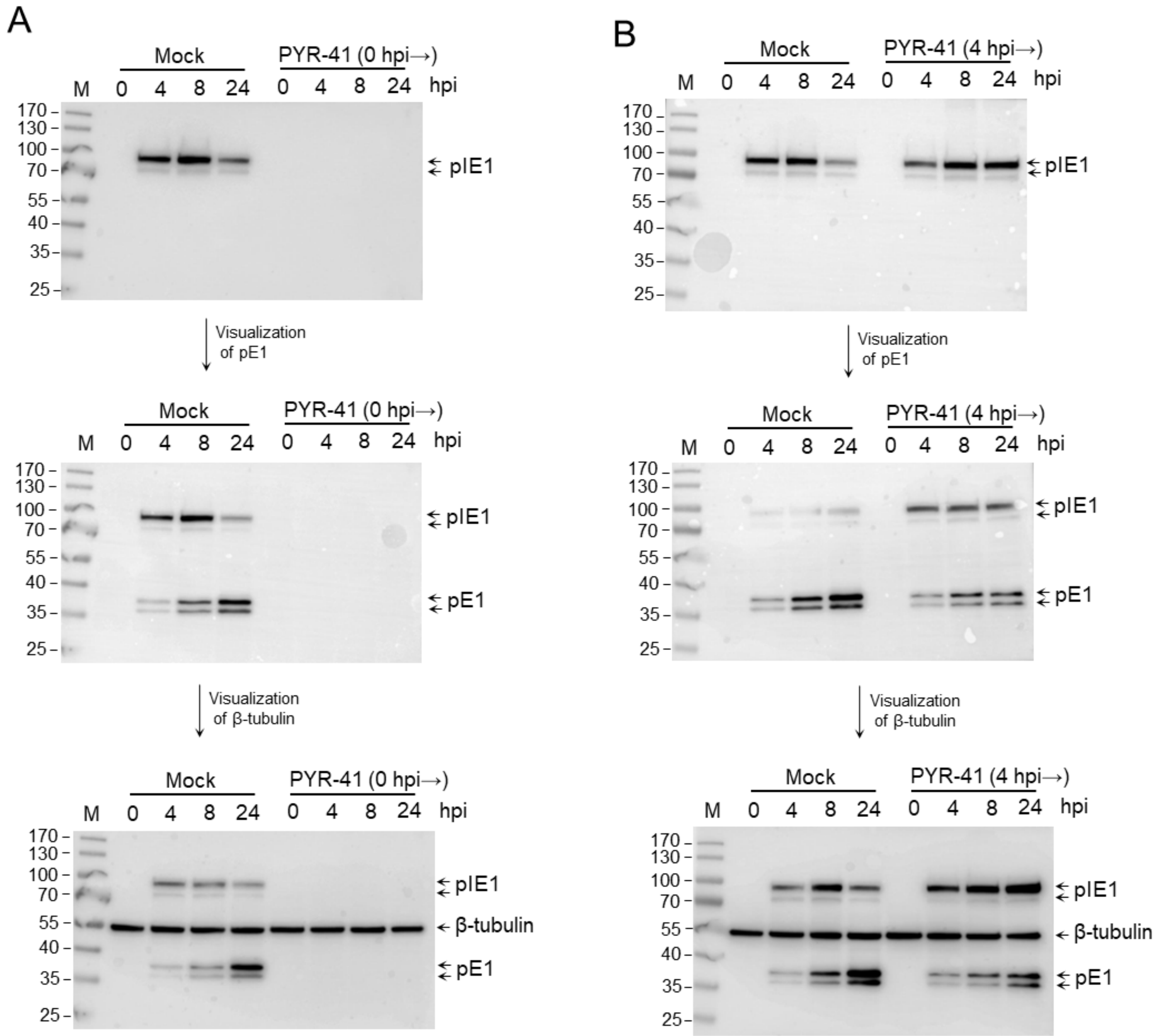

Figure S4. Complete Western blots related to Figure 1D and Figures 3A-B. NIH 3T3 cells were infected with wt MCMV (MOI of 10), and PYR41 (15 μM) was added at 0 hpi (**A**) or 4 hpi (**B**). Samples for Western blot analysis were lysed at the indicated time points. The expressions of pIE1, pE1 and β-tubulin were visualized stepwise with corresponding primary and secondary POD-conjugated antibodies and chemiluminescence.

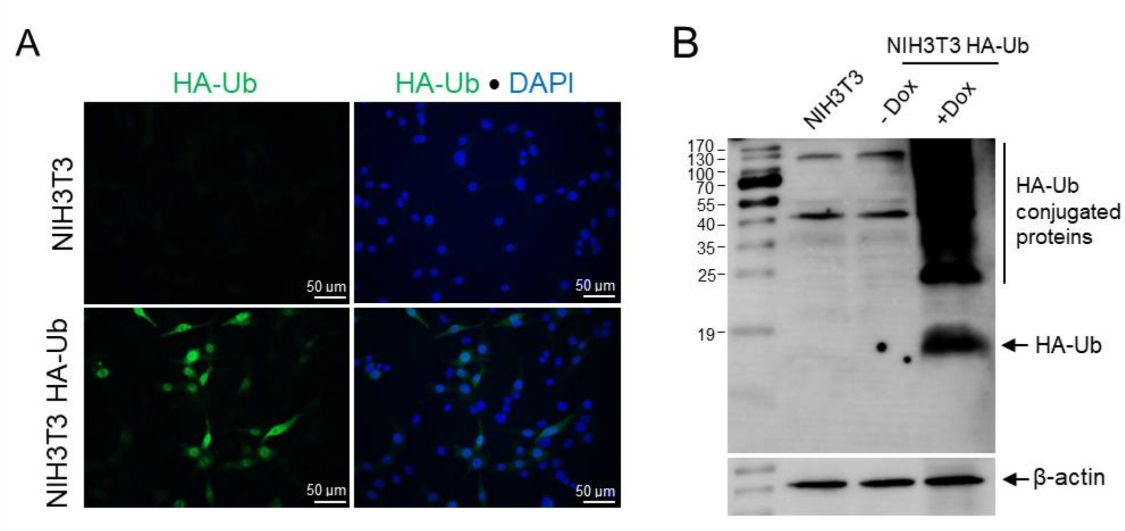

Figure S5. NIH3T3 HA-Ub cell lines with inducible expression of HA-Ub constructs. NIH3T3 HA-Ub cells were treated with doxycycline (Dox; 2 µg/ml) and analyzed after 48 hours. (**A**) HA-Ub on NIH3T3 HA-Ub cells were visualized with rabbit anti-HA pAb and AF^{488}-conjugated secondary rabbit antibody. (**B**) Visualization of HA-Ub (expected Mw~10.6 kDa) on NIH3T3 and NIH3T3 HA-Ub (with or without Dox) cells.

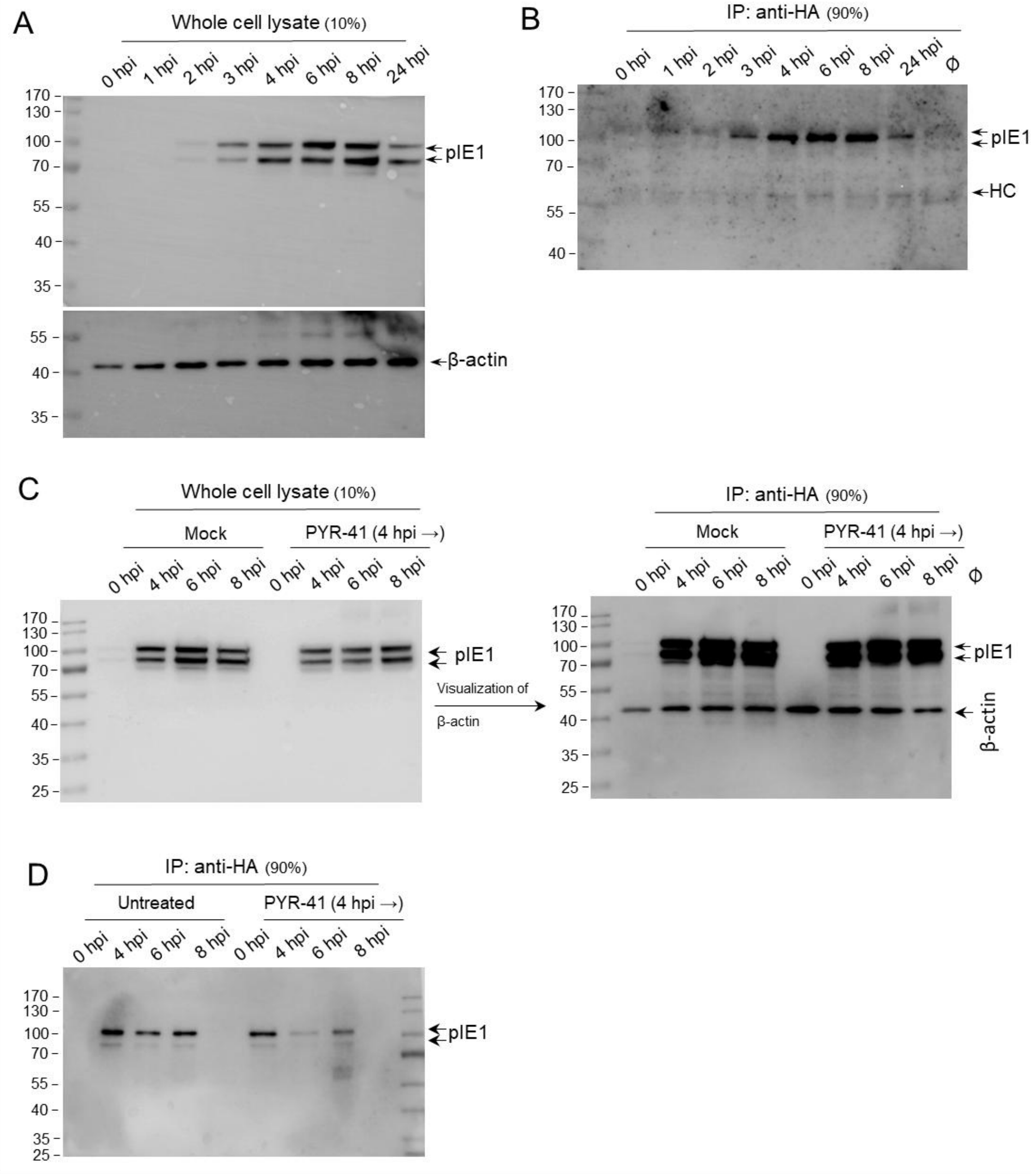

Figure S6. Complete Western blots related to Figure 2. (**A-B**) NIH3T3 HA-Ub cells were treated with doxycycline (2 µg/ml). After 48 hours, cells were infected with wt MCMV (MOI of 10), and at the indicated time points, (**A**) 10% of aliquots were lysed in RIPA for WCL and (**B**) 90% in 1% NP40 for IP of ubiquitinated proteins with rabbit anti-HA antibody and PAS. Expression of pIE1 and β-actin was visualized with corresponding primary and secondary POD-conjugated antibodies and chemiluminescence. (C) NIH3T3 HA-Ub cells were treated with doxycycline (2 µg/ml). After 48 hours, cells were infected with wt MCMV (MOI of 10) and 4 hours later treated with 15 µM PYR-41 or left untreated. At the indicated time points, cells were lysed and proceeded as described to obtain total (WCL) (**C**) or immunoprecipitated (**D**) pIE1.

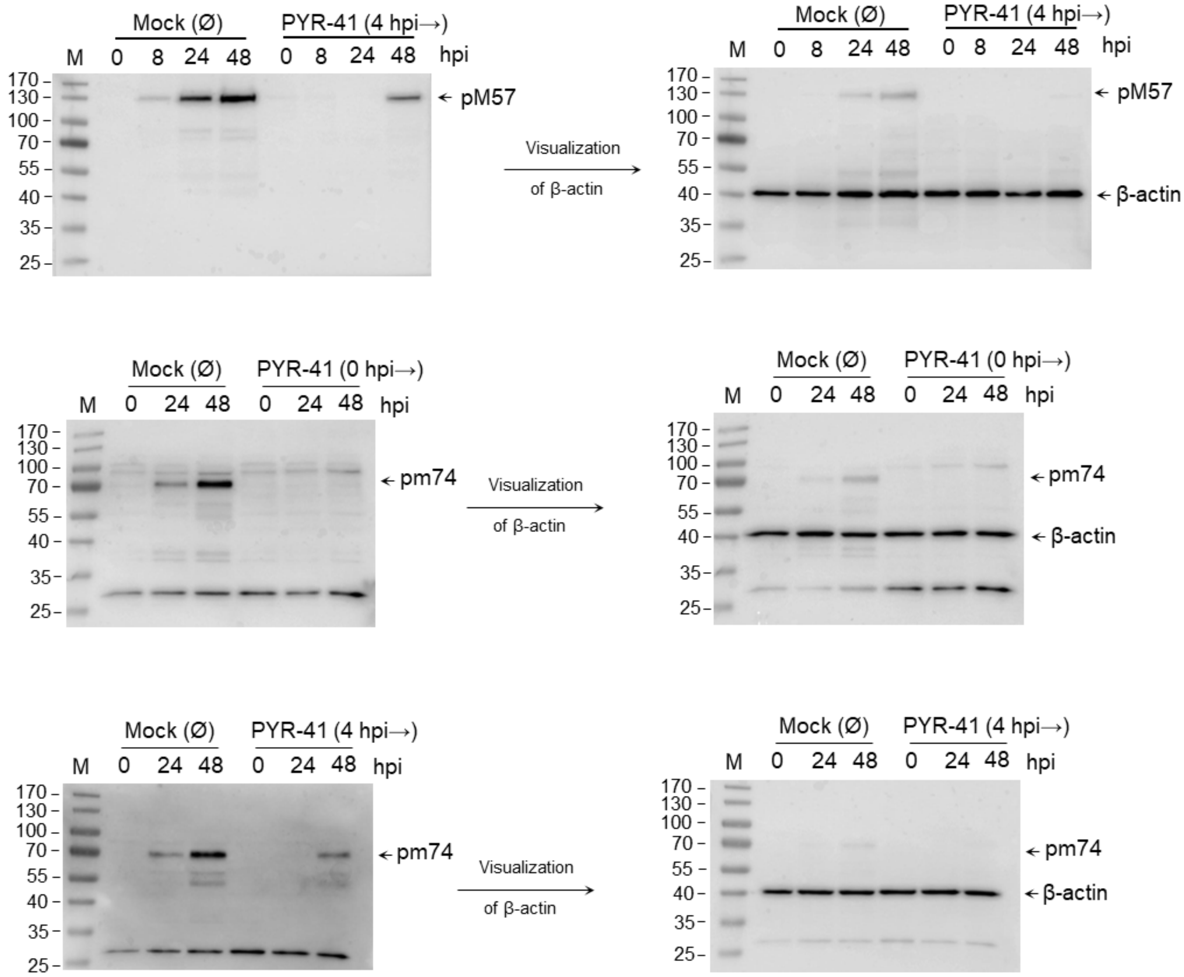

Figure S7. Complete Western blots related to Figure 3. PYR-41 affects the kinetics of early and late MCMV protein expression – complete western blots. NIH 3T3 cells were infected with wt MCMV (10 MOI), and PYR-41 (15 μM) was added 0 hpi or 4 hpi. Samples for Western blot analysis were lysed at the indicated time points. Expression of pM57 or pm74 MCMV proteins and β-tubulin or β-actin was visualized stepwise with corresponding primary and secondary POD-conjugated antibodies and chemiluminescence.

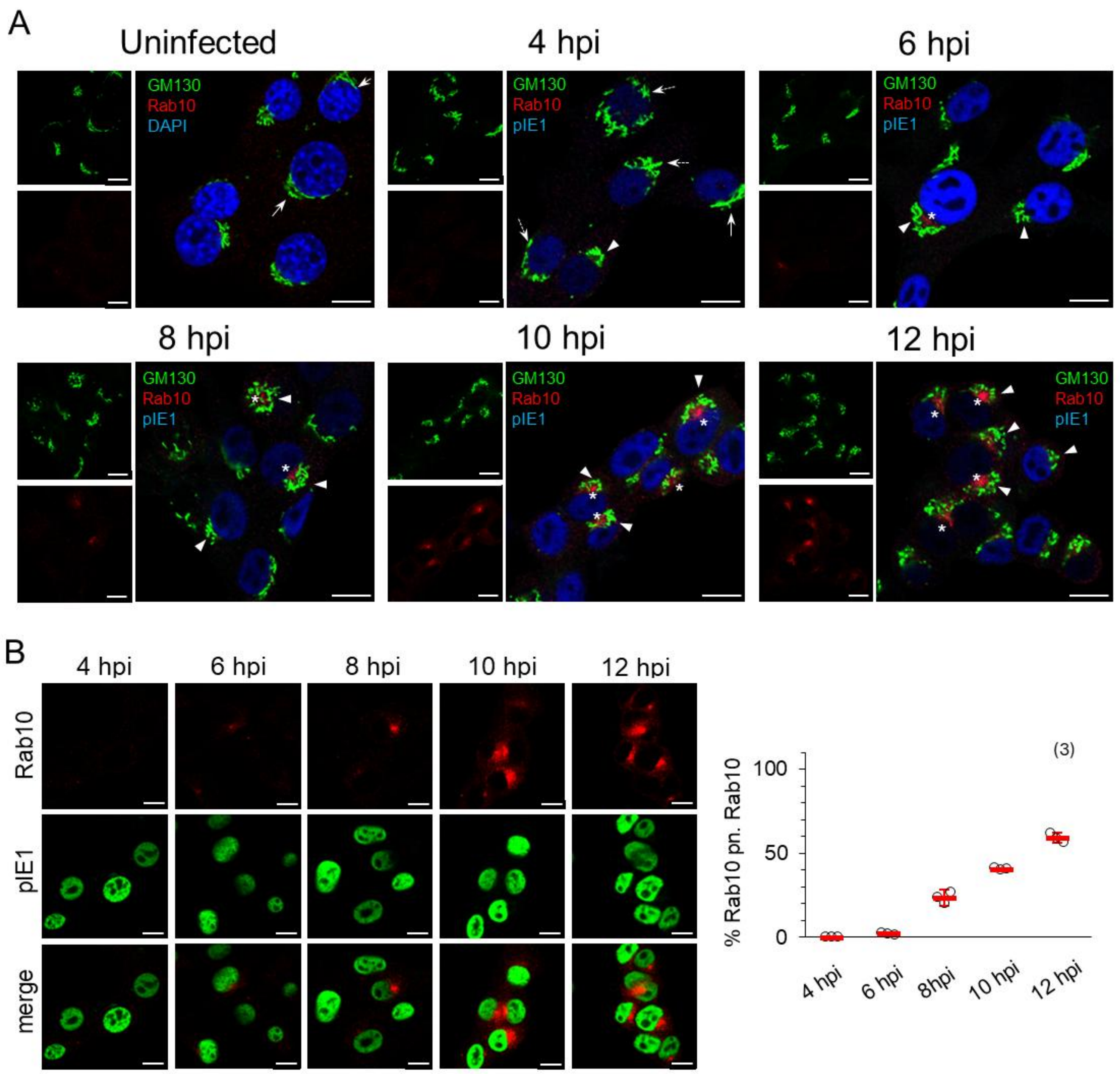

Figure S8. Kinetics of pre-AC formation in MCMV-infected NIH3T3 cells (related to Figure 4A). (**A**) NIH 3T3 cells were infected with ΔFcR-MCMV (MOI of 10), fixed and permeabilized at the indicated times post-infection (hpi). GM130 (green), Rab10 (red) and pIE1 (blue) were visualized with the corresponding primary and non-cross-reactive fluorescently labelled secondary antibodies. In uninfected cells, DAPI (blue) is used to visualize the nuclei. Arrows indicate extended Golgi (full arrows for normal Golgi cisternae and dashed arrows for unconnected/expanded Golgi cisternae), arrowheads indicate condensed Golgi, and asterisks indicate perinuclear Rab10 in pre-AC. (**B**) NIH3T3 cells were infected with wt MCMV (MOI of 10), fixed and permeabilized at the indicated times post-infection (hpi). Rab10 (red) and pIE1 (green) were visualized with the corresponding primary and non-cross-reactive fluorescently labelled secondary antibodies. The percentage of cells showing condensed perinuclear Rab10 signal in pre-AC is shown on the left. Data represents the mean ± S.D (red bars) and individual data (empty circles) from three independent experiments (indicated in parenthesis).

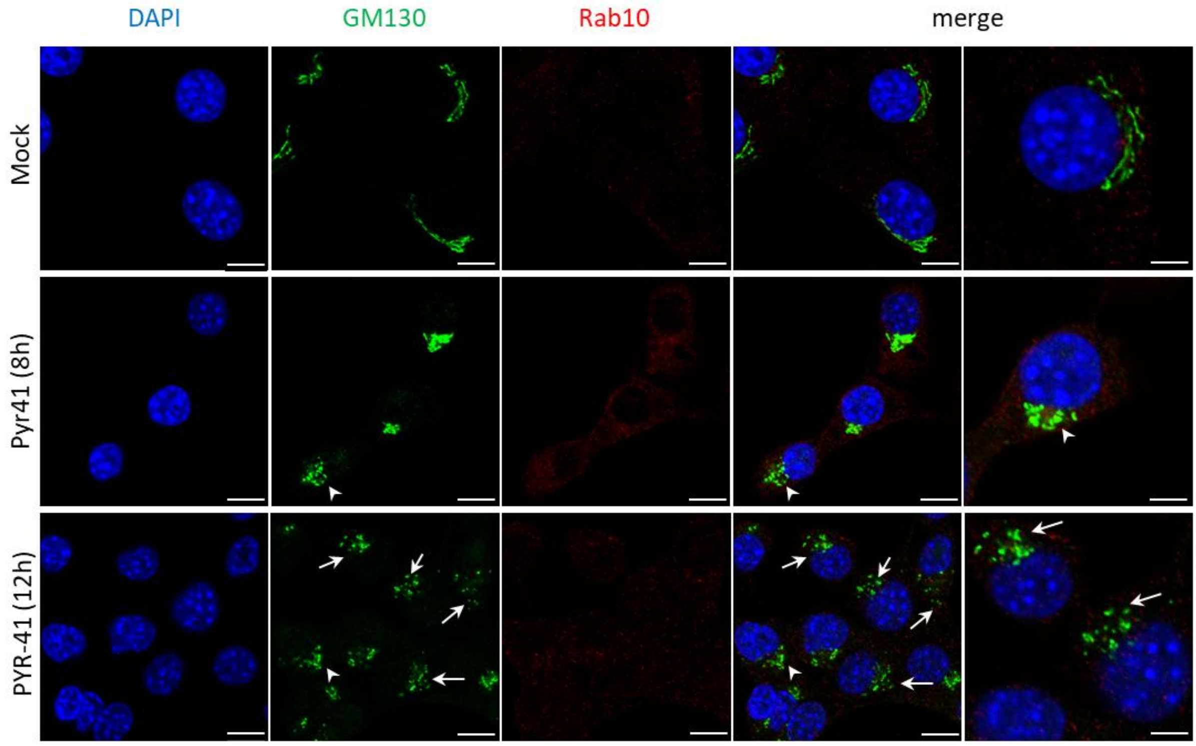

Figure S9. The effect of PYR-41 on the expression of Rab10 and GM130 in uninfected cells. NIH3T3 cells were treated with 15 μM PYR-41 for 8 or 12 hours or left untreated. After fixation and permeabilization, GM130 (green) and Rab10 (red) were visualized with corresponding primary and fluorescently labelled secondary antibodies. The nuclei were stained with DAPI (blue). Arrowheads indicate condensed Golgi and arrows indicate dispersed Golgi. Bars: 10 μm (lower magnification) and 5 μm (higher magnification).

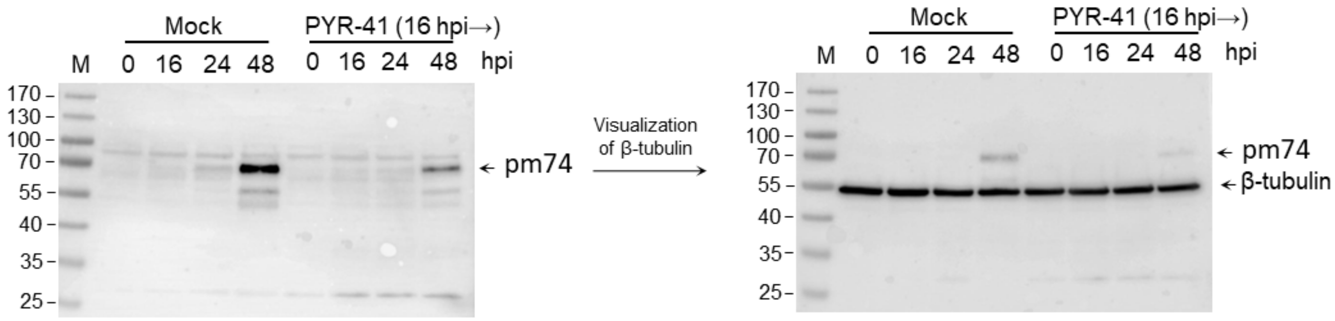

Figure S10. Complete Western blots related to Figure 6B. NIH 3T3 cells were infected with wt MCMV (MOI of 10) and treated with 15 μM PYR-41 at 16 hpi or left untreated. Samples for Western blot analysis were lysed at the indicated time points. The expression of pm74 MCMV protein and β-tubulin was visualized stepwise with corresponding primary and secondary POD-conjugated antibodies and chemiluminescence.

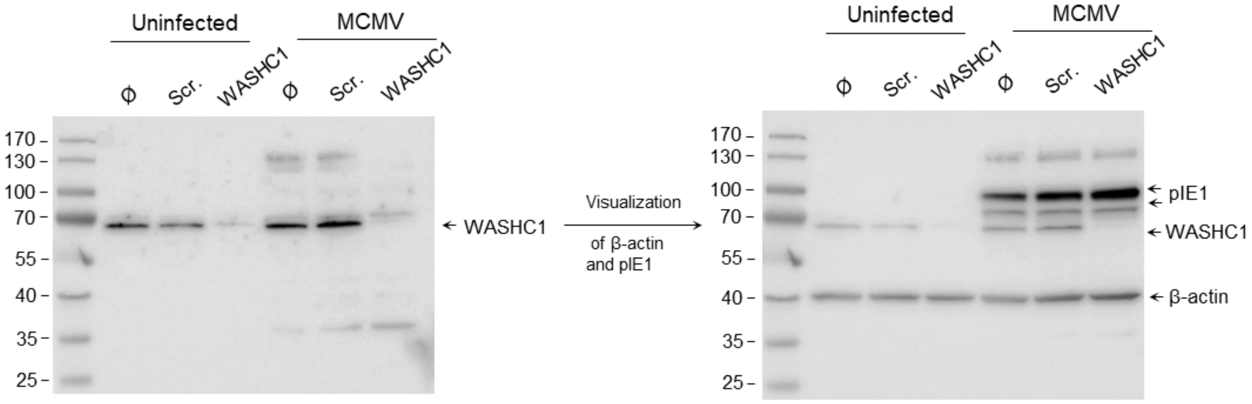

Figure S11. Complete Western blots related to Figure 9C. The expressions of WASHC1, pIE1, and β-tubulin were visualized stepwise with corresponding primary and secondary POD-conjugated antibodies and chemiluminescence.
